# Rhodoterpenoids A‒C, Three New Rearranged Triterpenoids from *Rhododendron latoucheae* by HPLC‒MS‒SPE‒NMR

**DOI:** 10.1038/s41598-017-06320-x

**Published:** 2017-08-11

**Authors:** Fei Liu, Ya-Nan Wang, Yong Li, Shuang-Gang Ma, Jing Qu, Yun-Bao Liu, Chang-Shan Niu, Zhong-Hai Tang, Tian-Tai Zhang, Yu-Huan Li, Li Li, Shi-Shan Yu

**Affiliations:** 1 State Key Laboratory of Bioactive Substance and Function of Natural Medicines, Institute of Materia Medica, Chinese Academy of Medical Sciences and Peking Union Medical College, Beijing, 100050 People’s Republic of China; 2 Institute of Medicinal Biotechnology, Chinese Academy of Medical Sciences and Peking Union Medical College, Beijing, 100050 People’s Republic of China

## Abstract

Rhodoterpenoids A‒C (**1**‒**3**), three new rearranged triterpenoids, together with one new biogenetically related compound, rhodoterpenoid D (**4**), were isolated and efficiently elucidated from *Rhododendron latoucheae* by high-performance liquid chromatography−mass spectrometry−solid-phase extraction−nuclear magnetic resonance (HPLC‒MS‒SPE‒NMR). Compounds **1** and **2** possess an unprecedented skeleton with a 5/7/6/6/6-fused pentacyclic ring system, while compound **3** contains a unique 6/7/6/6/6-fused pentacyclic carbon backbone. Their structures were determined by extensive spectroscopic methods and electronic circular dichroism (ECD) analyses. Plausible biogenetic pathways for **1**‒**4** were proposed. Compounds **1** and **4** showed potential activity against herpes simplex virus **1** (HSV-1) with IC_50_ values of 8.62 and 6.87 *μ*M, respectively.

## Introduction

The Ericaceae plants have high values in aesthetics and medicine with worldwide distribution. They contain a wide range of chemical components such as flavones^1^, diterpenes^2–4^, triterpenes^5^, phenols^6^, coumarins^7^, and lignans^8, 9^ which possess pharmacological activities that include anti-inflammatory^10^, analgesic^2–4^, anti-oxidant^11^, anti-bacterial^11^, anti-HIV^12^, immunity^13^, and cytotoxicity^14^ properties.

**Figure 1:**
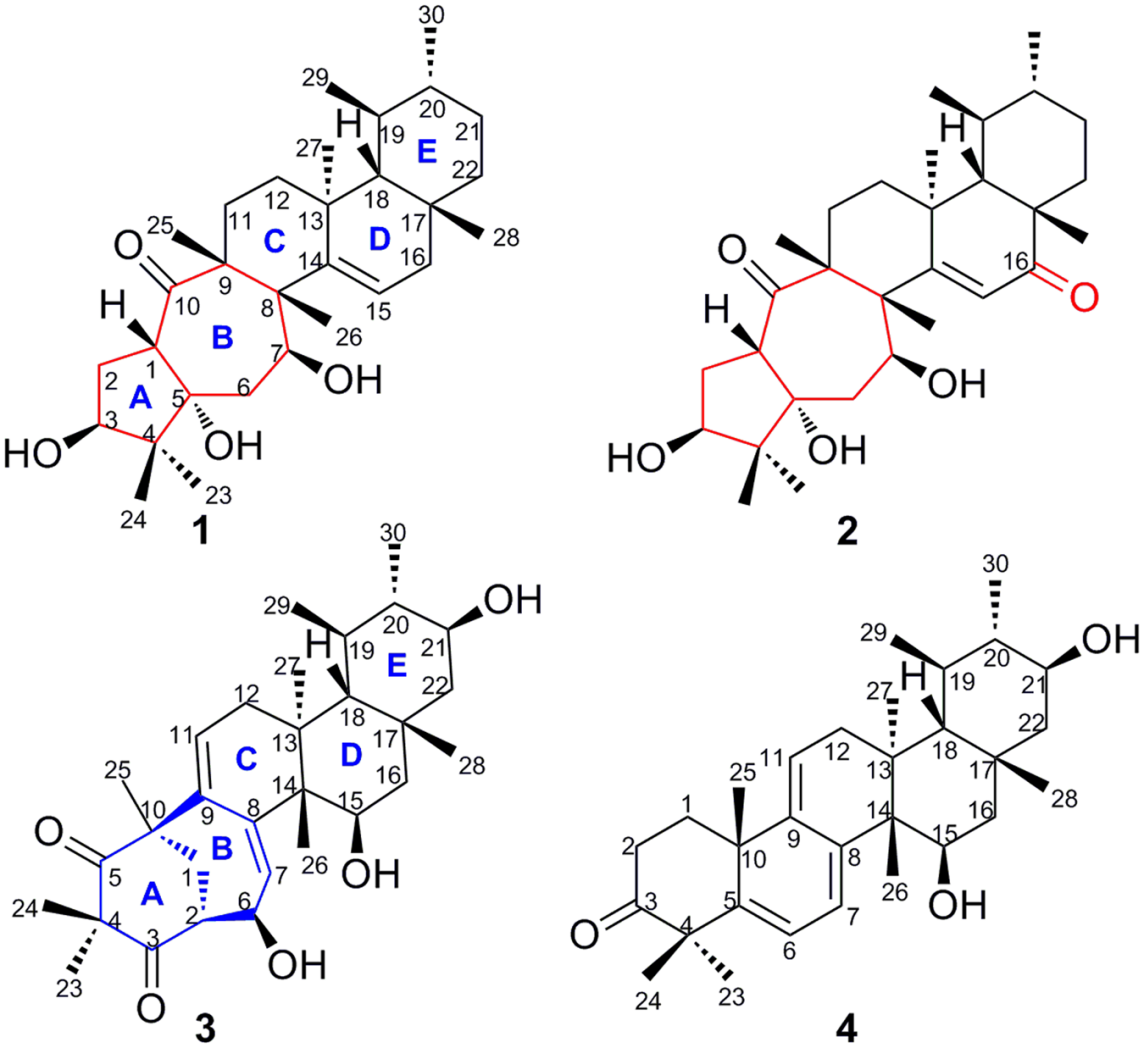
Structures of rhodoterpenoids A‒D (**1**‒**4**).

*Rhododendron latoucheae* Finet *et* Franch, a plant of the family Ericaceae, is mainly distributed in southern and southwestern mainland China. It has been historically used as a traditional folk medicine for its effects in eliminating phlegm, suppressing cough, activating blood and dissolving stasis^15^. To date, only two literature articles describing chemical investigations of this plant have been published, and mostly considered the phenols and iridoids^16, 17^. The previous work carried out by our group over the years led to the isolation of new active terpenoids from plants of the family Ericaceae^2–4, 18, 19^. Therefore, our research on this plant was mainly focused on the attractive terpenoids. A preliminary investigation of this plant indicated that the CH_2_Cl_2_-soluble fraction from *Rhododendron latoucheae* contained many minor triterpenoids that were extremely difficult to obtain by traditional methods of isolation. It is noteworthy that natural products have played an invaluable role in the drug discovery process, and the natural extracts contain many minor constituents, some of which have significant physiological and biological activity^20–23^. Here, the application of the hyphenated technique of HPLC‒MS‒SPE‒NMR^24, 25^, a method that makes the structural analysis of minor components of mixtures feasible, successfully overcame this challenge. Thus, through our further investigation of this plant for the identification of structurally unique and biologically interesting triterpenoids, rhodoterpenoids A‒D (**1**‒**4**) (Fig. 1) were isolated by the technology of HPLC‒MS‒SPE‒NMR. Notably, compounds **1** and **2** possess an unprecedented skeleton having a 5/7/6/6/6-fused pentacyclic ring system, while compound **3** contains a unique 6/7/6/6/6-fused pentacyclic carbon backbone. In addition, the new compound **4** obtained from the title plant in the present study was a key precursor before the rearrangement of rings A and B to obtain compound **3**. Herein, we report their isolation, structural elucidation, biological evaluation, and possible biogenetic pathways.

## Results and Discussion

**Figure 2:**
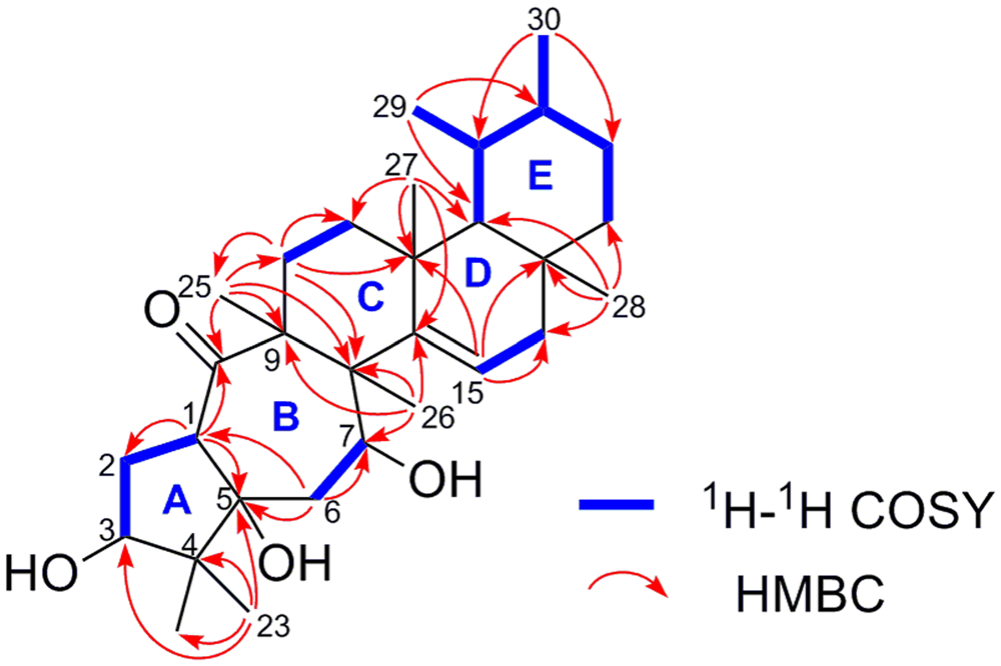
^1^H‒^1^H COSY and key HMBC correlations of (**1**).

Rhodoterpenoid A (**1**) was obtained as a white amorphous powder with the molecular formula C_30_H_48_O_4_, indicating seven degrees of unsaturation (Table 1). The ^1^H NMR spectrum of **1** [Table 2 and Figure S5 in the Supporting Information (SI)] displayed signals of two secondary methyls at *δ*_H_ 1.00 (d, *J* = 6.5 Hz, H_3_-30) and 1.11 (d, *J* = 7.1 Hz, H_3_-29), six tertiary methyls at *δ*_H_ 0.86 (H_3_-24), 0.94 (H_3_-23), 0.99 (H_3_-25), 1.08 (H_3_-28), 1.13 (H_3_-27), and 1.32 (H_3_-26), two oxygen-bearing methines at *δ*_H_ 4.04 (dd, *J* = 9.6 and 8.0 Hz, H-3) and 4.37 (dd, *J* = 10.9 and 3.7 Hz, H-7), and one olefinic proton at 5.78 (dd, *J* = 5.9 and 3.4 Hz, H-15). Its ^13^C NMR spectrum (Table 3 and Figure S6 in the SI) and DEPT experiment revealed eight methyls, seven methylenes, seven methines (one olefinic and two oxygenated), and eight quaternary carbons (one olefinic, one oxygenated, and one ketone). These data suggested a pentacyclic structure and also a triol should be present in **1**. The ^1^H-^1^H COSY spectrum (Figure S9 in the SI) revealed the presence of the spin-coupling systems shown in bold in Fig. 2. The HMBC correlations (Figure S8 in the SI) from H_3_-23 to C-3, C-4, C-5, and C-24, from H-1 to C-2, C-5, and C-10, and from H-6a to C-1, C-5, and C-7 allowed the five-membered ring (ring A in Fig. 2) to be defined. Subsequently, the seven-membered ring B fused with ring A was deduced from the HMBC cross-peaks of H_3_-25 with C-8, C-9, C-10, and C-11, and of H_3_-26 with C-7, C-8, C-9, and C-14 (Fig. 2). Finally, the common six-membered rings C, D and E were indicated by the HMBC correlations from H-11a to C-8, C-12, C-13, and C-25, from H-15 to C-13, C-16, and C-17, from H_3_-27 to C-12, C-13, C-14, and C-18, from H_3_-28 to C-16, C-17, C-18, and C-22, from H_3_-29 to C-18 and C-20, and from H_3_-30 to C-19 and C-21 (Fig. 2). Thus, the gross structure of rhodoterpenoid A was elucidated to be **1**, which possesses a remarkable 5/7/6/6/6 pentacyclic skeleton.

**Table 1:** The MS data of compounds **1**–**4**.

compounds	molecular formula	HRSIMS data (*m/z*)	calc for	unsaturation degrees
**1**	C30H48O4	495.3438 [M + Na]^+^	495.3445	7
**2**	C30H46O5	487.3428 [M + H]^+^	487.3418	8
**3**	C30H44O5	507.3081 [M + Na]^+^	507.3081	9
**4**	C30H44O3	453.3357 [M + H]^+^	453.3363	9

**Table 2:** ^1^H NMR (600 MHz) data of compounds **1**–**4** in CD_3_OD.

No.	1	2	3	4
	*δ*_H_ (*J* in Hz)	*δ*_H_ (*J* in Hz)	*δ*_H_ (*J* in Hz)	*δ*_H_ (*J* in Hz)
1a	3.71 (dd, 11.0, 5.8)	3.71 (dd, 11.0, 5.6)	2.70 (dd, 14.9, 5.7)	2.14 (m)
1b			2.17 (dd, 14.9, 1.9)	2.14 (m)
2a	2.86 (ddd, 13.9, 9.6, 5.8)	2.87 (ddd, 15.9, 9.6, 5.6)	2.97 (m)	2.62 (m)
2b	1.42 (m)	1.44 (m)		2.62 (m)
3	4.04 (dd, 9.6, 8.0)	4.04 (dd, 9.5, 8.2)		
6a	2.05 (dd, 13.9, 3.7)	2.02 (m)	4.41 (dd, 3.8, 2.5)	5.91 (d, 6.2)
6b	1.94 (dd, 13.9, 10.9)	2.02 (m)		
7	4.37 (dd, 10.9, 3.7)	4.51 (dd, 10.5, 4.2)	5.64 (brs)	6.17 (d, 6.2)
11a	2.30 (ddd, 14.0, 9.4, 7.6)	2.36 (td, 12.9, 11.9, 3.3)	5.75 (dd, 3.8, 3.7)	5.57 (m)
11b	1.63 (m)	1.79 (m)		
12a	1.59 (m)	1.75 (m)	2.01 (m)	2.04 (m)
12b	1.59 (m)	1.75 (m)	2.01 (m)	2.04 (m)
15	5.78 (dd, 5.9, 3.4)	5.98 (s)	4.01 (dd, 11.1, 5.7)	4.10 (dd, 10.1, 6.7)
16a	2.15 (dd, 17.7, 3.4)		1.44 (m)	1.51 (m)
16b	1.99 (dd, 17.7, 5.9)		1.44 (m)	1.51 (m)
18	1.24 (d, 2.1)	1.73 (m)	1.32 (d, 2.2)	1.35 (brs)
19	1.37 (m)	1.33 (s)	1.17 (m)	1.15 (m)
20	1.53 (m)	1.61 (m)	1.54 (m)	1.56 (m)
21a	1.60 (m)	1.67 (m)	3.44 (ddd, 11.7, 9.8, 6.6)	3.44 (ddd, 11.4, 9.7, 6.6)
21b	1.21 (m)	1.28 (m)		
22a	1.39 (m)	1.72 (m)	2.15 (m)	2.21 (dd, 14.9, 9.7)
22b	1.56 (m)	1.72 (m)	1.18 (m)	1.21 (m)
23	0.94 (s)	0.94 (s)	1.24 (s)	1.26 (s)
24	0.86 (s)	0.87 (s)	1.22 (s)	1.29 (s)
25	0.99 (s)	1.01 (s)	1.38 (s)	1.11 (s)
26	1.32 (s)	1.40 (s)	0.77 (s)	1.05 (s)
27	1.13 (s)	1.28 (s)	0.65 (s)	0.70 (s)
28	1.08 (s)	1.31 (s)	1.17 (s)	1.20 (s)
29	1.11 (d, 7.1)	1.17 (d, 6.8)	1.14 (d, 6.6)	1.14 (d, 6.7)
30	1.00 (d, 6.5)	1.00 (d, 6.5)	1.06 (d, 6.1)	1.06 (d, 6.1)

**Table 3:** ^13^C NMR (150 MHz) data of compounds **1**–**4** in CD_3_OD.

No.	1	2	3	4
1	56.4 (d)	56.7 (d)	36.9 (t)	30.9 (t)
2	30.4 (t)	30.2 (t)	52.0 (d)	35.3 (t)
3	78.2 (d)	78.0 (d)	214.9 (s)	217.4 (s)
4	51.6 (s)	51.6 (s)	60.2 (s)	50.4 (s)
5	82.6 (s)	82.6 (s)	215.9 (s)	151.4 (s)
6	39.3 (t)	41.3 (t)	74.5 (d)	119.3 (d)
7	68.0 (d)	68.3 (d)	129.9 (d)	118.9 (d)
8	51.6 (s)	53.2 (s)	143.6 (s)	139.3 (s)
9	54.7 (s)	54.6 (s)	140.1 (s)	144.7 (s)
10	215.3 (s)	214.5 (s)	48.9 (s)	40.4 (s)
11	30.1 (t)	29.5 (t)	126.1 (d)	120.3 (d)
12	35.0 (t)	33.5 (t)	40.2 (t)	39.3 (t)
13	40.0 (s)	40.9 (s)	43.0 (s)	42.3 (s)
14	148.7 (s)	177.7 (s)	49.1 (s)	46.5 (s)
15	128.3 (d)	129.3 (d)	65.9 (d)	67.2 (d)
16	42.9 (t)	208.9 (s)	47.5 (t)	47.9 (t)
17	33.1 (s)	43.7 (s)	36.0 (s)	35.9 (s)
18	60.8 (d)	60.3 (d)	53.0 (d)	52.9 (d)
19	36.4 (d)	36.6 (d)	36.2 (d)	36.3 (d)
20	35.5 (d)	33.4 (d)	41.2 (d)	41.2 (d)
21	29.6 (t)	29.2 (t)	73.0 (d)	73.0 (d)
22	36.8 (t)	30.7 (t)	44.0 (t)	44.3 (t)
23	19.1 (q)	19.0 (q)	27.3 (q)	28.8 (q)
24	18.0 (q)	18.0 (q)	20.5 (q)	27.2 (q)
25	24.1 (q)	24.0 (q)	27.3 (q)	26.8 (q)
26	18.8 (q)	18.6 (q)	10.6 (q)	12.2 (q)
27	22.4 (q)	23.4 (q)	19.3 (q)	18.2 (q)
28	37.4 (q)	33.4 (q)	38.0 (q)	38.3 (q)
29	27.0 (q)	25.6 (q)	25.5 (q)	25.4 (q)
30	22.9 (q)	22.5 (q)	19.0 (q)	18.8 (q)

**Figure 3:**
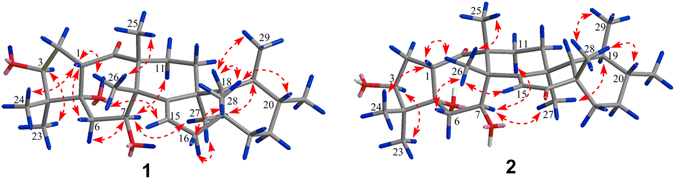
Key NOE correlations for (**1**) and (**2**).

The relative configuration of **1** was elucidated by nuclear Overhauser effect (NOE) experiments (Fig. 3 and Figure S10 in the SI). The NOE correlations of H-3/H_3_-23, H-1/H_3_-24, H-1/H-6b, H-6a/H-7/H-11a, and H-7/H_3_-27/H-16a suggested that H-3, H-7, H_3_-23, H_3_-27 and OH-5 were cofacial and *α*-oriented. In addition, the NOE correlations of H-1/H_3_-26/H_3_-25, H_3_-26/H-15, H-16b/H_3_-28/H-20, H-19/H_3_-27, and H_3_-28/H-18/H_3_-29 confirmed that H-1, H-18, H-20, H_3_-24, H_3_-25, H_3_-26, H_3_-28 and H_3_-29 were on the same side with a *β* -direction.

**Figure 4:**
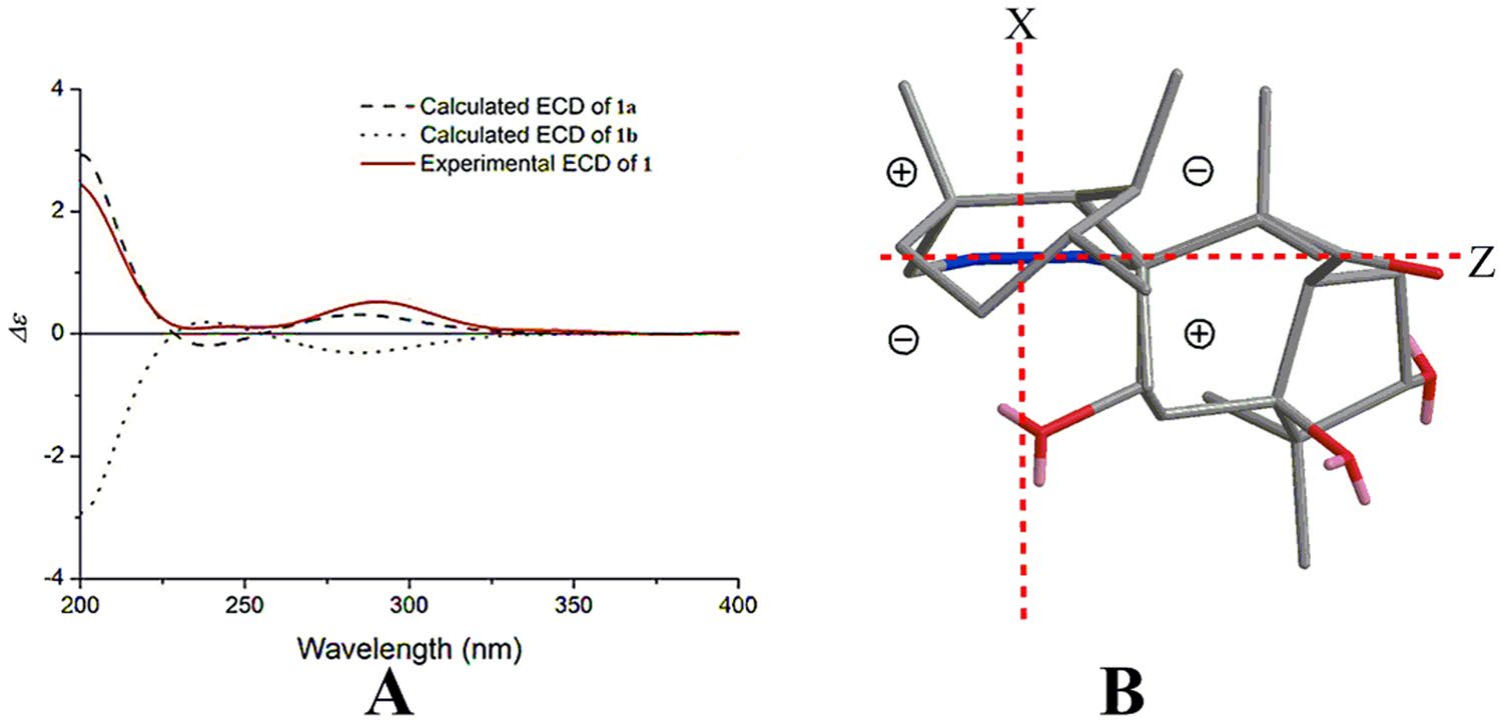
(**A**) Experimental ECD spectrum of **1**, using TDDFT at the B3LYP/6-31 G(d) level in MeOH calculated ECD spectra of **1a** and **1b**. (**B**) Application for the olefin octant rule of **1** (rear octants viewed along Y-axis).

Based on the above results, there were only two possible structures for **1**, with absolute configurations of **1a** (1 *S*, 3 *S*, 5 *S*, 7 *S*, 8 *S*, 9 *R*, 13 *S*, 17 *R*, 18 *R*, 19 *S*, 20 *R*) and **1b** (1 *R*, 3 *R*, 5 *R*, 7 *R*, 8 *R*, 9 *S*, 13 *R*, 17 *S*, 18 *S*, 19 *R*, 20 *S*). Thus, the electronic circular dichroism (ECD) spectra of **1a** and its enantiomer **1b** were calculated using TDDFT. The experimental ECD spectrum of **1** was in good agreement with the calculated ECD of **1a** and was the opposite of **1b** (Fig. 4A), which suggested an absolute configuration of 1 *S*, 3 *S*, 5 *S*, 7 *S*, 8 *S*, 9 *R*, 13 *S*, 17 *R*, 18 *R*, 19 *S* and 20 *R* for compound **1**. This absolute configuration of **1** was substantiated by the olefin octant rule (Fig. 4B). The experimental ECD spectrum of **1** showed a positive Cotton effect near 200 nm, corresponding to the *π* → π* electronic transition of the Δ14 double bond^26, 27^.

Rhodoterpenoid B (**2**) was assigned a molecular formula C_30_H_46_O_5_ on the basis of its (+)-HRESIMS data (Table 1). A comparison of the ^1^H and ^13^C NMR data of **2** with those of **1** (Tables 2 and 3) revealed that they possessed the same carbon skeleton and implied a C-16 carbonyl in **2** instead of a methylene in **1** (C-16: *δ*_C_ 208.9 for **2**, *δ*_C_ 42.9 for **1**). This deduction was confirmed by the HMBC correlations (Figure S18 in the SI) of H_3_-28 with C-16, of H-15 with C-16, of H-22 with C-16, and of H-18 with C-16. The key NOE correlations (Fig. 3 and Figure S20 in the SI) of H-3/H_3_-23, H_3_-24/H-1/H_3_-26/H-6, H-7/H-11a, H-7/H_3_-27/H-19, H-15/H_3_-26/H_3_-25, and H-20/H_3_-28/H_3_-29 confirmed that the relative configuration of **2** was absolutely identical to that of **1**.

**Figure 5:**
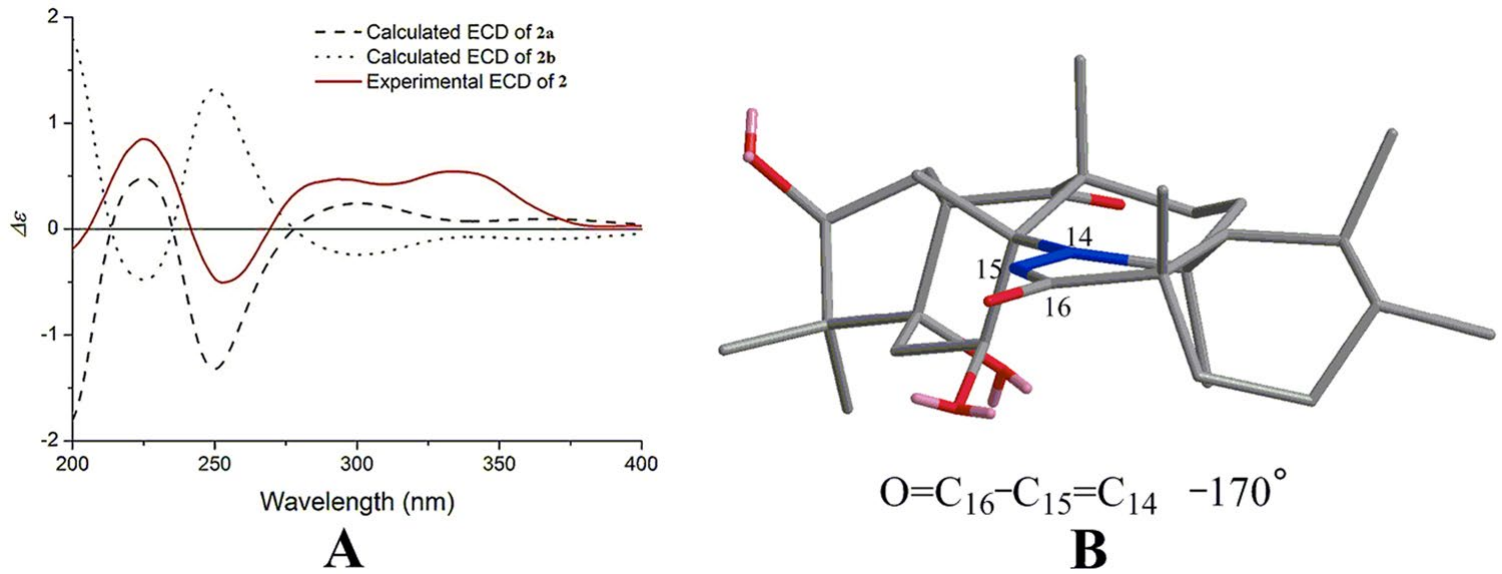
(**A**) Experimental ECD spectrum of **2**, using TDDFT at the B3LYP/6-31 G(d) level in MeOH calculated ECD spectra of **2a** and **2b**. (**B**) Application for the helicity rule of **2**.

The absolute configuration of compound **2**, was established identical as that of **1** by the same procedure of ECD spectra calculation of both enantiomers. The experimental ECD spectrum of **2** agreed well with the one calculated for **2a** (1 *S*, 3 *S*, 5 *S*, 7 *S*, 8 *S*, 9 *R*, 13 *S*, 17 *S*, 18 *S*, 19 *S*, 20 *R*) and was the opposite of **2b** (Fig. 5A). The absolute configuration of compound **2** was further substantiated by applying the helicity rule^28, 29^ for the *α*,*β*-unsaturated ketone with a negative Cotton effect at 253 nm (Fig. 5B).

**Figure 6:**
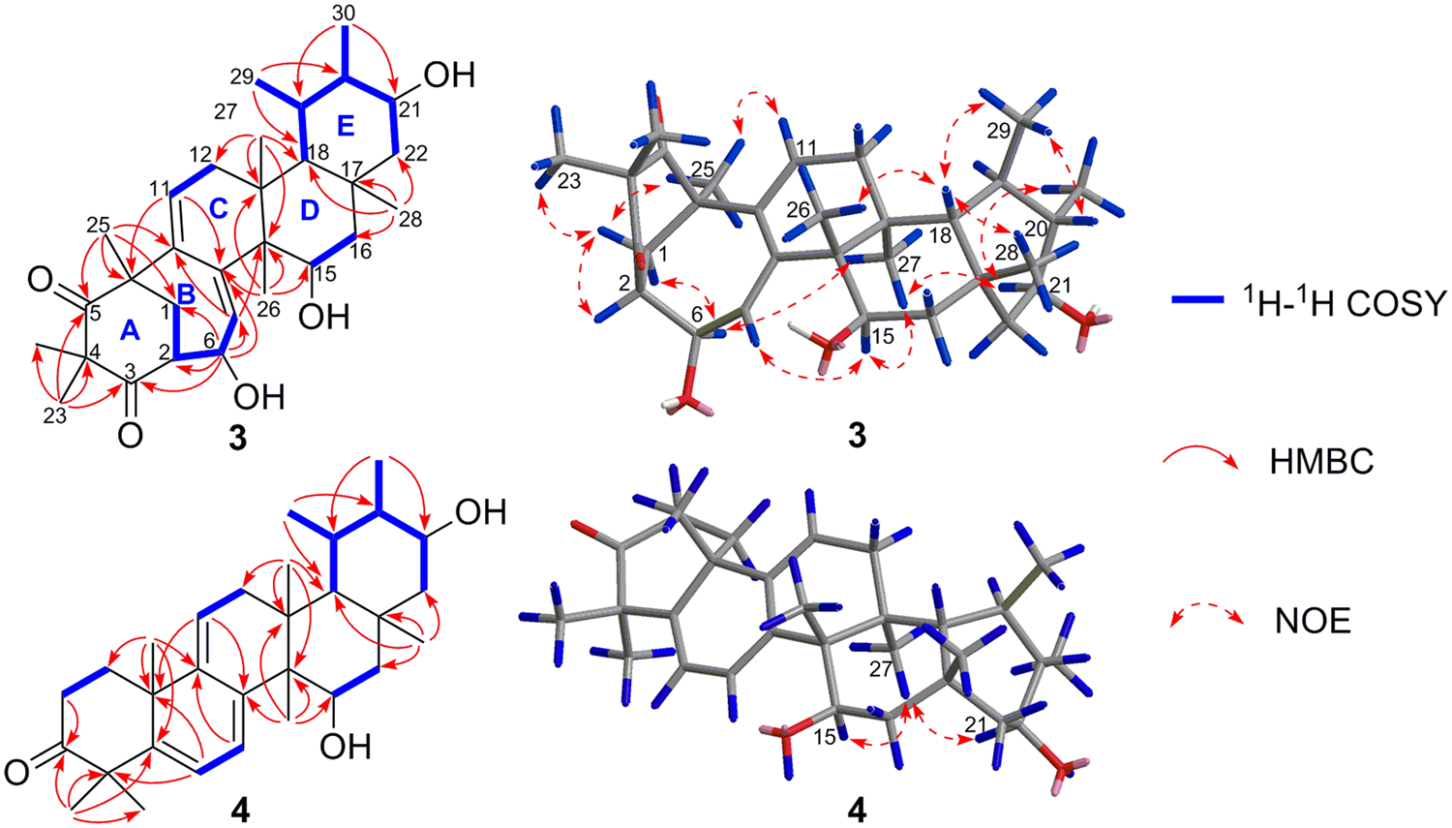
Selected ^1^H-^1^H COSY, HMBC, and NOE correlations for (**3**) and (**4**).

Rhodoterpenoid C (**3**) was assigned a molecular formula of C_30_H_44_O_5_ by its HRESIMS data (Table 1). The ^1^H NMR spectrum of **3** (Table 2 and Figure S25 in the SI) showed signals for two secondary methyls at *δ*_H_ 1.06(d, *J* = 6.1 Hz, H_3_-30) and 1.14 (d, *J* = 6.6 Hz, H_3_-29), six tertiary methyls at *δ*_H_ 0.65 (H_3_-27), 0.77 (H_3_-26), 1.17 (H_3_-28), 1.22 (H_3_-24), 1.24 (H_3_-23), and 1.38 (H_3_-25), three oxygen-bearing methines at *δ*_H_ 3.44 (ddd, *J* = 11.7, 9.8 and 6.6 Hz, H-21), 4.01 (dd, *J* = 11.1 and 5.7 Hz, H-15), and 4.41 (dd, *J* = 3.8 and 2.5 Hz, H-6), and two olefinic proton at 5.64 (brs, H-7) and 5.75 (dd, *J* = 3.8 and 3.7 Hz, H-11). Its ^13^C NMR spectrum (Table 3 and Figure S26 in the SI) and DEPT experiment disclosed eight methyls, four methylenes, nine methines (two olefinic and three oxygenated), and nine quaternary carbons (two olefinic and two ketones). Thus, compound **3** was also a pentacyclic triterpenoid and four aditional degrees of unsaturation described in the above data. The ^1^H-^1^H COSY spectrum (Figure S29 in the SI) suggested the presence of the spin-coupling systems shown in bold in Fig. 6. The HMBC correlations (Figure S28 in the SI) from H_3_-23 to C-3, C-4, C-5, and C-24, from H_3_-25 to C-1, C-5, C-9, and C-10, and from H-6 to C-1, C-2, C-3, C-7, and C-8 allowed the six-membered carbon ring (ring A in Fig. 6) to be defined. Then, the seven-membered ring B sharing C-1, C-2, and C-10 with ring A was deduced from the HMBC cross-peaks of H-7 with C-9 and C-14 and of H-11 with C-8 and C-10 (Fig. 6). Finally, the common six-membered rings C, D and E were indicated by the HMBC correlations from H_3_-26 to C-8, C-13, C-14, and C-15, from H_3_-27 to C-12, C-13, C-14, and C-18, from H_3_-28 to C-16, C-17, C-18, and C-22, from H_3_-29 to C-18, and C-20, and from H_3_-30 to C-19, and C-21 (Fig. 6). Therefore, the gross structure of rhodoterpenoid C was elucidated to be **3**, which contains a unique 6/7/6/6/6-fused pentacyclic carbon backbone. The relative configuration of **3** was deduced from NOE experiments. The NOE correlations (Fig. 6 and Figure S30 in the SI) of H_3_-23/H-1a/H-2, H-1a/H_3_-25/H-11, H-1b/H-6/H_3_-27/H-21/H_3_-30, and H-7/H-15/H_3_-27 showed that H-2, H-6, H-15, H-21, H_3_-23, H_3_-25 and H_3_-27 were cofacial and *α*-oriented. In addition, the NOE correlations (Fig. 6) of H_3_-26/H-18/H_3_-28 and H-18/H_3_-29/H-20 indicated that H-18, H-20, H_3_-26, H_3_-28, and H_3_-29 were on the same side with a *β* -direction.

**Figure 7:**
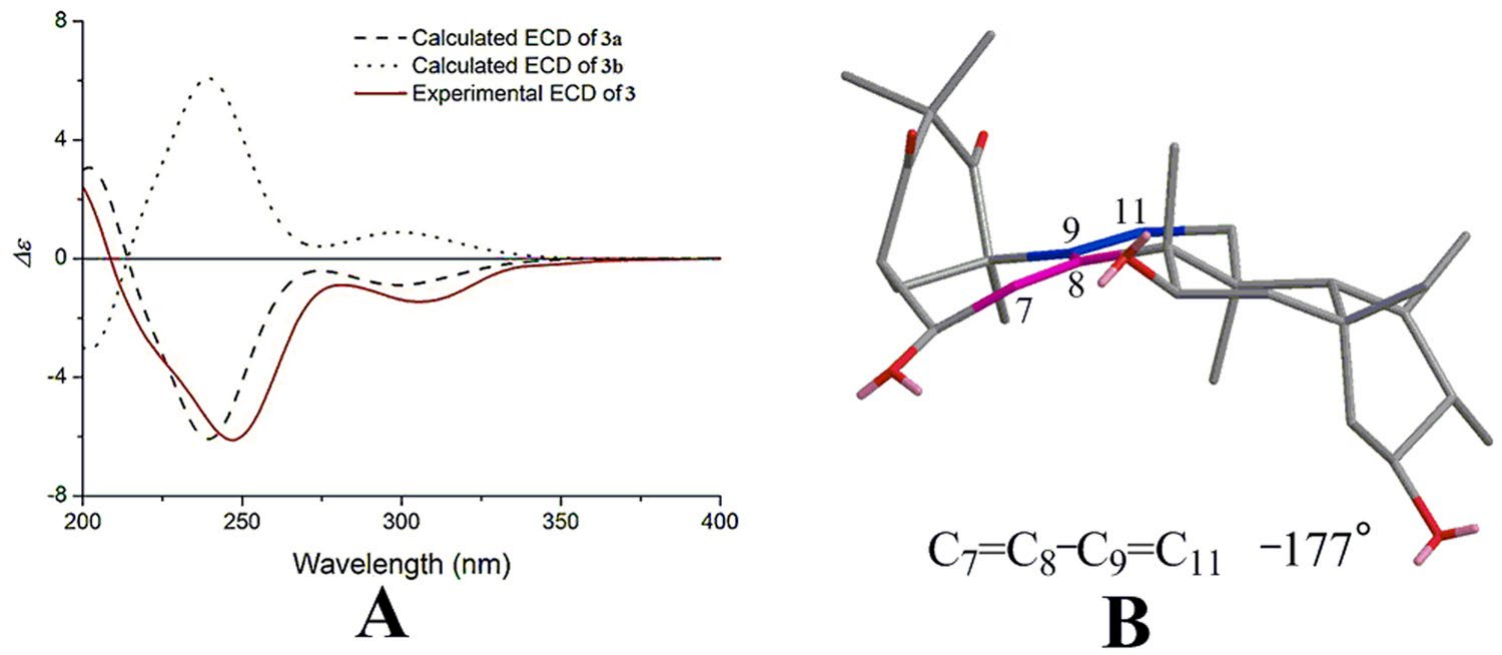
(**A**) Experimental ECD spectrum of **3**, using TDDFT at the B3LYP/6-31 G(d) level in MeOH calculated ECD spectra of **3a** and **3b**. (**B**) Application for the helicity rule of **3**.

As previously, there were only two possible structures for 3 (3a and 3b), and the absolute configuration was established via experimental and calculated ECD (after failed attempts to obtain a single crystal of **3**). Again, the experimental ECD spectrum of **3** correlated fairly well with the calculated ECD of **3a** and was the opposite of that of **3b** (Fig. 7A), which suggested an absolute configuration of 2 *S*, 6 *R*, 10 *R*, 13 *S*, 14 *S*, 15 *R*, 17 *R*, 18 *R*, 19 *R*, 20 *S* and 21 *S* for compound **3**. The negative Cotton effect at 247 nm (Fig. 7B) by applying the helicity rule for non-planar *transoid* dienes^30^ further demonstrated this absolute configuration of **3**.

Rhodoterpenoid D (**4**) was assigned a molecular formula of C_30_H_44_O_3_ by its HRESIMS data (Table 1). Extensive analysis of 1D (Tables 2 and 3) and 2D NMR spectra revealed that **4** possessed a bauerene skeleton. The proposed structure of **4** was fully determined by its HSQC, ^1^H−^1^H COSY, and HMBC spectra (Fig. 6). The key NOE correlations (Fig. 6) of H-15/H_3_-27/H-21 confirmed that H-15 and H-21 were on the same side with a *α*-direction, and its absolute configuration was finally deduced as 10 *R*, 13 *S*, 14 *S*, 15 *R*, 17 *R*, 18 *R*, 19 *R*, 20 *S* and 21 *S* by applying the octant rule for the saturated ketone^31^. The plane projection of optimized conformation of **4** with the above absolute configuration on the rear octants showed the negative sign, which agreed well with the negative Cotton effect at 312 nm in the experimental ECD spectrum.

**Figure 8:**
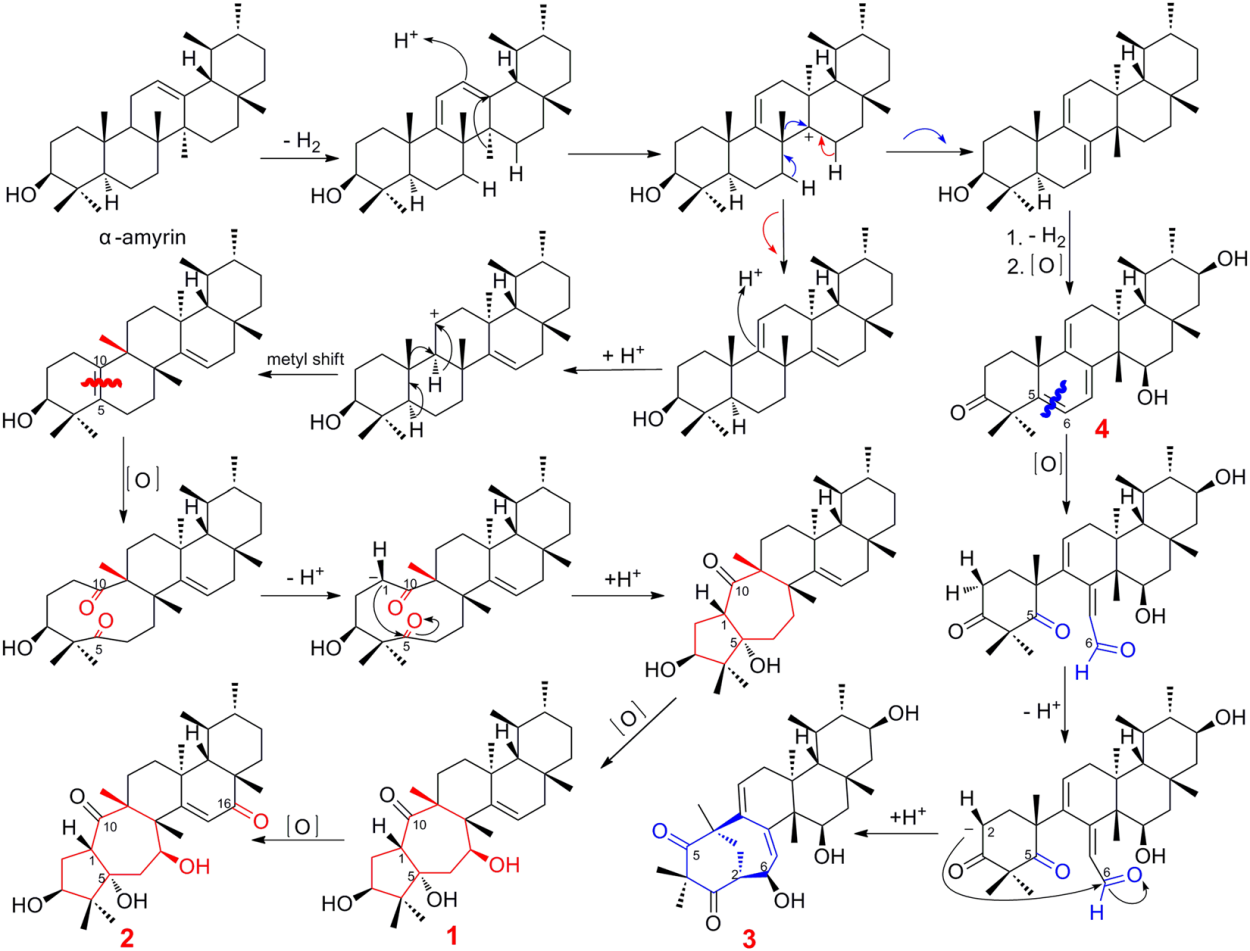
Plausible Biogenetic Pathway of (**1**‒**4**).

A plausible biogenetic pathway for rhodoterpenoids A‒D (**1**‒**4**) was proposed as shown in Fig. 8. Rhodoterpenoids A‒C (**1**‒**3**) are novel rearranged triterpenoids that may be derived from *α*-amyrin, which is a common triterpene occurring in natural plant populations^32^. As shown, *α*-amyrin underwent several dehydrogenations, hydrogenations, oxidations, methyl shifts, ring-opening reactions by double-bond oxidation and cyclizations to afford compounds **1**‒**3**. It is noteworthy that the isolation of new compound **4**, which was a key precursor before the rearrangement of rings A and B to obtain compound **3**, rationalized the proposed biogenetic pathway for compound **3**.

The compounds were tested for their anti-inflammatory and anti-virus activities, because some studies have shown that the pentacyclic triterpenoids have these activities^33–36^. Compounds **1**‒**4** were evaluated *in vitro* for anti-inflammatory activity using the measurement of the inhibition of lipopolysaccharide (LPS)-induced TNF-*α* production in RAW264.7 cells by enzyme-linked immunosorbent assay^37^ with dexamethasone as a positive control. Compounds **2**‒**4** showed relatively weak inhibitory effects, with inhibition of TNF-*α* production at 29%, 7.9% and 30%, respectively, at a concentration of 10 *μ*M. Because of the scarce amounts obtained of compound **3**, only compounds **1**, **2** and **4** were tested for antiviral [herpes simplex virus-1 (HSV-1)] activity. Compounds **1** and **4** showed potential activity against HSV-1, with IC_50_ values of 8.62 and 6.87 *μ*M, respectively, and compound **2** was inactive (Table 4). The above data implied that replacement of the methylene at C-16 in **1** with a ketone carbon dramatically decreased anti-HSV-1 activity in **2**.

**Table 4:** Antiviral activity against HSV-1 and cytotoxicity for compounds **1, 2**, and **4** in Vero cells^*a*^.

compound	TC_50_^*b*^ (*μ*M)	IC_50_ (*μ*M)	SI^*c*^
**1**	19.25	8.62	2.2
**2**	69.34	>33.33	—^*d*^
**4**	48.07	6.87	7.0
Acyclovir^*e*^	>100	0.41	>243.9

^*a*^Data represent mean values for three independent determinations; ^*b*^Cytotoxic concentration required to inhibit Vero cell growth by 50%; ^*c*^Selectivity index value equaled TC_50_/IC_50_; ^*d*^The selectivity index (SI) could not be determined under the test conditions; ^*e*^Positive control.

In summary, four new triterpenoids, including two with an unprecedented 5/7/6/6/6-fused pentacyclic ring system and one with a unique 6/7/6/6/6-fused pentacyclic carbon backbone, were rapidly obtained and efficiently elucidated from *Rhododendron latoucheae* by HPLC‒MS‒SPE‒NMR. Their absolute configurations were determined by ECD analyses combined with experimental rules^26–31^ after unsuccessful attempts to obtain single crystals of **1**‒**3**. It is significant that the minor novel compounds were isolated and efficiently elucidated by the hyphenated technique of HPLC‒MS‒SPE‒NMR. These newly discovered compounds not only provide new challenges and opportunities for synthesis and biological evaluation but also prompt us to pay more attention to the triterpenes of the Ericaceae plants.

## Materials and Methods

### General experimental procedures

Optical rotations were measured with a JASCO P-2000 automatic digital polarimeter. UV spectra were measured on a JASCO V650 spectrophotometer. CD spectra were recorded on a JASCO-815 CD spectrometer. IR spectra were measured on a Nicolet 5700 FT-IR microscope instrument. HPLC–MS–SPE–NMR were carried out by using a chromatographic separation system consisted of an Agilent 1260 series HPLC with an in-line solvent degasser, quaternary pump, auto-sampler, column compartment with thermostat, and a diode array detector. The chromatographic separation was carried out using a YMC-Pack Pro C_18_ column (4.6 mm × 250 mm, 5*μ*m), and the column temperature was maintained at 40 °C. ESIMS were performed using a Bruker micrOTOF-Q II, while NMR spectra were obtained on a Bruker AVANCE III HD 600 MHz spectrometer except for that the NOE spectra of compounds **1** and **4** were measured on an Agilent-NMR-vnmrs 600 spectrometer. Chemical shifts are given in *δ* (ppm) with the solvent (CD_3_OD; *δ*_H_ 3.31; *δ*_C_ 49.0) peaks used as references. (+)-HRESIMS data of compounds **1**–**3** were recorded using an Agilent 6520 Accurate-Mass Q-TOF LC/MS spectrometer, and (+)-HRESIMS data of compound **4** was performed on a Bruker micrOTOF-Q II. Polyamide resin (30−60 mesh, Jiangsu Linjiang Chemical Reagents Factory, Linjiang, China), macroporous resin (D101 type, Chemical Plant of Nankai University, Nankai, China), MCI gel (Mitsubishi Chemical Corporation), Sephadex LH-20 (GE Chemical Corporation), Silica gel (200–300 mesh, Qingdao Marine Chemical Factory, China), and ODS (50 *μ*m, Merck, Germany) were used for column chromatography (CC). TLC was carried out with glass precoated Silica gel GF_254_ plates (Qingdao Marine Chemical Factory, China). Spots were visualized by spraying with 10% H_2_SO_4_ in EtOH followed by heating.

### Plant material

Twigs and leaves of *Rhododendron latoucheae* were collected from Zhangjiajie, Hunan Province, People’s Republic of China, in October 2014 and identified by Prof. Lin Ma of the Chinese Academy of Medical Sciences and Peking Union Medical College. A voucher specimen (ID-22815) was deposited in the herbarium at the Department of Medicinal Plants, Institute of Materia Medica, Chinese Academy of Medical Sciences.

### Extraction and isolation

The air-dried twigs and leaves of *Rhododendron latoucheae* (107 kg) were extracted with 95% EtOH/H_2_O (2 h × 3; 10 L/Kg) under reflux conditions. The crude extract (6000 g) was suspended in 30 L of H_2_O and then partitioned with petroleum ether, CH_2_Cl_2_, EtOAc and n-butanol (3 × 30 L). The CH_2_Cl_2_-soluble fraction (500 g) was subjected to a MCI gel column eluted with MeOH‒H_2_O (90:10, 100:0 v/v). The 90% MeOH fraction (350 g) was then further separated over a silica gel column and eluted in a gradient of petroleum ether/(Me)_2_CO (50:1, 30:1, 20:1, 10:1, 5:1, 1:1 v/v) to afford Fr.1‒Fr.7. Fr.4 (40 g) was applied to a Sephadex LH-20 column (petroleum ether/CH_2_Cl_2_/MeOH, 5:5:1) to obtain Fr.4-1 (25 g), which was further resolved on a Sephadex LH-20 column eluted with MeOH to get four subfractions (Fr.4-1-1‒Fr.4-1-4). Fr.4-1-3 (20 g) was subjected to MCI gel column eluted using a gradient of MeOH-H_2_O (30:70, 60:40, 70:30, 80:20, 100:0 v/v) to produce eleven subfractions (Fr.4-1-3 A‒Fr.4-1-3 K). Fr.4-1-3E (800 mg) was chromatographed over Sephadex LH-20 and ODS gel columns to produce five subfractions (Fr.4-1-3E4-1‒Fr.4-1-3E4-5). Fr.4-1-3E4-5 (20 mg) was purified by HPLC–MS–SPE–NMR with MeCN/H_2_O/TFA (45:55:0.055, v/v/v, 1.0 mL/min) to yield compound **3** (1.5 mg, t_R_ = 17.7 min) and **2** (2 mg, t_R_ = 20.1 min). Fr.4-1-3I (5 g) was subjected to silica gel, Sephadex LH-20 and ODS gel columns to produce Fr.4-1-3I4-1-5 (35 mg), which was further purified by HPLC–MS–SPE–NMR with MeOH/H_2_O/TFA (75:25:0.025, v/v/v, 1.0 mL/min) to afford compound **1** (2 mg, t_R_ = 50.3 min). Similarly, Fr.4-1-3 H (1 g) was subjected to Sephadex LH-20 and ODS gel columns to produce Fr.4-1-3H2-3 (20 mg), which was subsequently purified by HPLC–MS–SPE–NMR with MeCN/H_2_O/TFA (50:50:0.05, v/v/v, 1.0 mL/min) to obtain compound **4** (2 mg, t_R_ = 43.3 min).

**Rhodoterpenoid A (1)** white amorphous powder; ${[\alpha ]}_{D}^{20}$ + 38.9 (c 0.1, MeOH); UV (MeOH) *λ*_max_ (log *ε*): 203 (3.68) nm; CD (MeOH) max (Δε) 290 (+0.52) nm; IR (KBr) ν_max_: 3398, 2927, 2868, 1685, 1464, 1380, 1079, 1050, 1024, 976, 883, 837, 803, 724 cm^−1^; ^1^H NMR (CD_3_OD, 600 MHz) data, see Table 2; ^13^C NMR (CD_3_OD, 150 MHz) data, see Table 3; (+)-HRESIMS *m/z* 495.3438 [M + Na]^+^ (calcd for C_30_H_48_O_4_Na, 495.3445).

**Rhodoterpenoid B (2)**. white amorphous powder; ${[\alpha ]}_{D}^{20}$ + 19.6 (c 0.13, MeOH); UV (MeOH) *λ*_max_ (log *ε*): 244 (3.39) nm; CD (MeOH) max (Δε) 225 (+0.85), 253 (−0.5), 292 (+0.47), 334 (+0.54) nm; IR (KBr) ν_max_: 3394, 2957, 2933, 2872, 1686, 1640, 1459, 1380, 1307, 1249, 1182, 1094, 1071, 1049, 1027, 975, 914, 838, 802, 711, 630 cm^−1^; ^1^H NMR (CD_3_OD, 600 MHz) data, see Table 2; ^13^C NMR (CD_3_OD, 150 MHz) data, see Table 3; (+)-HRESIMS *m/z* 487.3428 [M + H]^+^ (calcd for C_30_H_47_O_5_, 487.3418).

**Rhodoterpenoid C (3)**. white powder; ${[\alpha ]}_{D}^{20}$ – 79.9 (c 0.04, MeOH); UV (MeOH) *λ*_max_ (log *ε*): 236 (3.56), 203 (3.56) nm; CD (MeOH) max (Δε) 247 (−6.11), 305 (−1.45) nm; IR (KBr) ν_max_: 3423, 2931, 2871, 1687, 1464, 1378, 1270, 1050, 1016, 903, 639 cm^−1^; ^1^H NMR (CD_3_OD, 600 MHz) data, see Table 2; ^13^C NMR (CD_3_OD, 150 MHz) data, see Table 3; (+)-HRESIMS *m/z* 507.3081 [M + Na]^+^ (calcd for C_30_H_44_O_5_Na, 507.3081).

**Rhodoterpenoid D (4)**. colorless oil; ${[\alpha ]}_{D}^{20}$ – 121.8 (c 0.07, MeOH); UV (MeOH) *λ*_max_ (log *ε*): 318 (3.46), 306 (3.44), 206 (3.74) nm; CD (MeOH) max (Δε) 208 (+10.08), 312 (−6.81) nm; IR (KBr) ν_max_: 3428, 2957, 1709, 1462, 1380, 1249,1077, 1040, 1014, 930, 905, 661 cm^−1^; ^1^H NMR (CD_3_OD, 600 MHz) data, see Table 2; ^13^C NMR (CD_3_OD, 150 MHz) data, see Table 3; (+)-HRESIMS *m/z* 453.3357 [M + H]^+^ (calcd for C_30_H_45_O_3_, 453.3363).

### Methodology for ECD calculation and experimental rules of **1**–**3**

Conformational analysis of **1**–**3** was performed using the MMFF94 molecular mechanics force field by MOE software and the conformers were optimized at B3LYP/6-31 G(d) level. The 50 lowest electronic transitions for the optimized conformations of **1**–**3** in MeOH were calculated using TDDFT method, and their theoretical ECD spectra were afforded by a Gaussian function with a half-bandwidth of 0.35 eV. The application of experimental rules for compounds **1**–**4** were based on their only optimized conformations obtained by the above method.

### Anti-inflammatory assay

RAW264.7 cells were cultured in 96-well plates (1 × 10^4^ cell mL^−1^) and preincubated with compounds for 1 h, followed by a further 12 h treatment with LPS for measurement of TNF-*α*. TNF-*α* content in the culture medium were measured by ELISA using anti-mouse TNF-*α* antibodies and a biotinylated secondary antibody, according to the manufacturer’s instructions. ELISA kit was obtained from Invitrogen by Thermo Fisher Scientific(Catalog numbr:88-7324). The optical density of each well was measured at 450 nm with an ELISA reader (Synergy H1, BioTeck, VT, USA). The RAW264.7 cells were obtained from the American Type Culture Collection (ATCC).

### Antiviral assays

African green monkey kidney cells (Vero) were from the Institute of Virology, Chinese Academy of Preventive Medicine^38^. Herpes simplex virus-1 (HSV-1 F strain VR 733) were obtained from the American Type Culture Collection (ATCC).

### Cytotoxic assay

The cytotoxicity of compounds **1**, **2**, and **4** in the presence of Vero cells were monitored by cytopathic effect (CPE). Vero cells (2.5 × 10^4^/well) were plated into a 96-well plate. A total of 24 h later, the monolayer cells were incubated with various concentrations of test compounds. After 48 h of culture at 37 °C and 5% CO_2_ in a carbon-dioxide incubator, the cells were monitored by CPE. Median toxic concentration (TC_50_) was calculated by Reed and Muench analyses.

### Anti-HSV-1 assay

The anti-HSV-1 activity of the compounds **1**, **2**, and **4** was assayed by the CPE inhibition method. Vero cells (2.5 × 10^4^cells/well) were plated into 96-well culture plates for an incubation period of 24 h. The cells were infected with 100 *μ*L of HSV-1 at 100TCID50 for 2 h at 37 °C. Then, various concentrations of the test compounds were added after removed the medium. Viral cytopathic effect (CPE) was observed when the CPE of the control group cells reached a value of 4+. Each experiment was performed in triplicate at least three separate times. The IC_50_ value is defined as the minimal concentration of inhibitor required to inhibit 50% of the CPE, as determined by the Reed and Muench method. The selectivity index was calculated as the ratio of TC_50_/IC_50_.

## Electronic supplementary material

Supplementary information
