# Supplementary material for: Rhodoterpenoids A‒C, Three New Rearranged Triterpenoids from Rhododendron latoucheae by HPLC‒MS‒SPE‒NMR
Source: Sci Rep. 2017 Aug 11;7:7944. doi: 10.1038/s41598-017-06320-x (PMC5554136; doi:10.1038/s41598-017-06320-x)
Supplement: Supplementary file 1 — Supplementary information [file 41598_2017_6320_MOESM1_ESM.pdf]

Supporting Information

Rhodoterpenoids A–C, Three New Rearranged Triterpenoids from *Rhododendron latoucheae* by HPLC–MS–SPE–NMR

Fei Liu[1], Ya-Nan Wang[1], Yong Li[1], Shuang-Gang Ma[1], Jing Qu[1], Yun-Bao Liu[1], Chang-Shan Niu[1], Zhong-Hai Tang[1], Tian-Tai Zhang[1], Yu-Huan Li[2], Li Li[1], Shi-Shan Yu[1,*]

[1] State Key Laboratory of Bioactive Substance and Function of Natural Medicines, Institute of Materia Medica, Chinese Academy of Medical Sciences and Peking Union Medical College, Beijing 100050, People's Republic of China.

[2] Institute of Medicinal Biotechnology, Chinese Academy of Medical Sciences and Peking Union Medical College, Beijing 100050, People's Republic of China.

*Corresponding author Tel.: +86-10-63165326. Fax: +86-10-63017757.
E-mail: yushishan@imm.ac.cn.

List of Contents

Contents		page
**Figure S1.**	The UV spectrum of compound **1** in MeOH	3
**Figure S2.**	The experimental ECD spectrum of compound **1** in MeOH	3
**Figure S3.**	The IR spectrum of compound **1**	4
**Figure S4.**	The (+)-HRESIMS data of compound **1**	4
**Figure S5.**	The ^{1}H NMR spectrum of compound **1** in CD_3OD	5
**Figure S6.**	The ^{13}C NMR spectrum and DEPT spectrum of compound **1** in CD_3OD	5
**Figure S7.**	The HSQC spectrum of compound **1** in CD_3OD	6
**Figure S8.**	The HMBC spectrum of compound **1** in CD_3OD	6
**Figure S9.**	The ^{1}H-^{1}H COSY spectrum of compound **1** in CD_3OD	7
**Figure S10.**	The NOE spectrum of compound **1** in CD_3OD	7-8
**Figure S11.**	The UV spectrum of compound **2** in MeOH	9
**Figure S12.**	The experimental ECD spectrum of compound **2** in MeOH	9
**Figure S13.**	The IR spectrum of compound **2**	10
**Figure S14.**	The (+)-HRESIMS data of compound **2**	10
**Figure S15.**	The ^{1}H NMR spectrum of compound **2** in CD_3OD	11
**Figure S16.**	The ^{13}C NMR spectrum and DEPT spectrum of compound **2** in CD_3OD	11
**Figure S17.**	The HSQC spectrum of compound **2** in CD_3OD	12
**Figure S18.**	The HMBC spectrum of compound **2** in CD_3OD	12
**Figure S19.**	The ^{1}H-^{1}H COSY spectrum of compound **2** in CD_3OD	13
**Figure S20.**	The NOE spectrum of compound **2** in CD_3OD	13-14
**Figure S21.**	The UV spectrum of compound **3** in MeOH	15
**Figure S22.**	The experimental ECD spectrum of compound **3** in MeOH	15
**Figure S23.**	The IR spectrum of compound **3**	16
**Figure S24.**	The (+)-HRESIMS data of compound **3**	16
**Figure S25.**	The ^{1}H NMR spectrum of compound **3** in CD_3OD	17
**Figure S26.**	The ^{13}C NMR spectrum and DEPT spectrum of compound **3** in CD_3OD	17
**Figure S27.**	The HSQC spectrum of compound **3** in CD_3OD	18
**Figure S28.**	The HMBC spectrum of compound **3** in CD_3OD	18
**Figure S29.**	The ^{1}H-^{1}H COSY spectrum of compound **3** in CD_3OD	19
**Figure S30.**	The NOE spectrum of compound **3** in CD_3OD	19-20
**Figure S31.**	The UV spectrum of compound **4** in MeOH	21
**Figure S32.**	The experimental ECD spectrum of compound **4** in MeOH	21
**Figure S33.**	The IR spectrum of compound **4**	22
**Figure S34.**	The (+)-HRESIMS data of compound **4**	22
**Figure S35.**	The ^{1}H NMR spectrum of compound **4** in CD_3OD	23
**Figure S36.**	The ^{13}C NMR spectrum and DEPT spectrum of compound **4** in CD_3OD	23
**Figure S37.**	The HSQC spectrum of compound **4** in CD_3OD	24
**Figure S38.**	The HMBC spectrum of compound **4** in CD_3OD	24
**Figure S39.**	The ^{1}H-^{1}H COSY spectrum of compound **4** in CD_3OD	25
**Figure S40.**	The NOE spectrum of compound **4** in CD_3OD	25

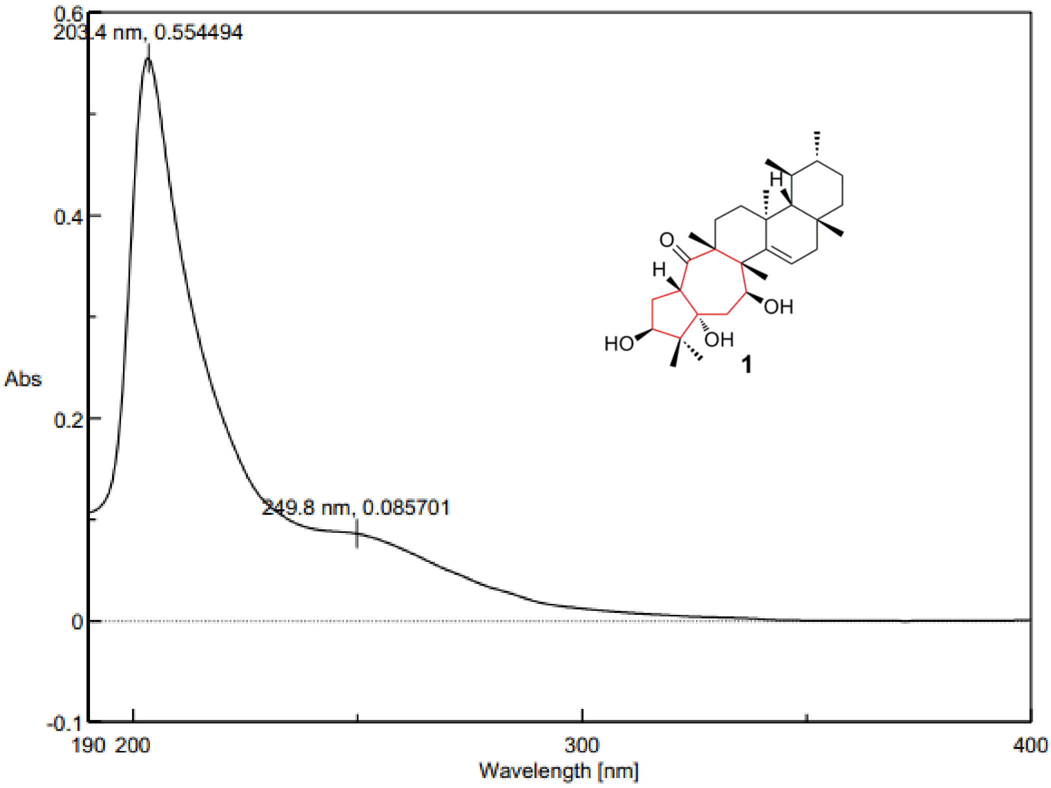

Figure S1. The UV spectrum of compound **1** in MeOH

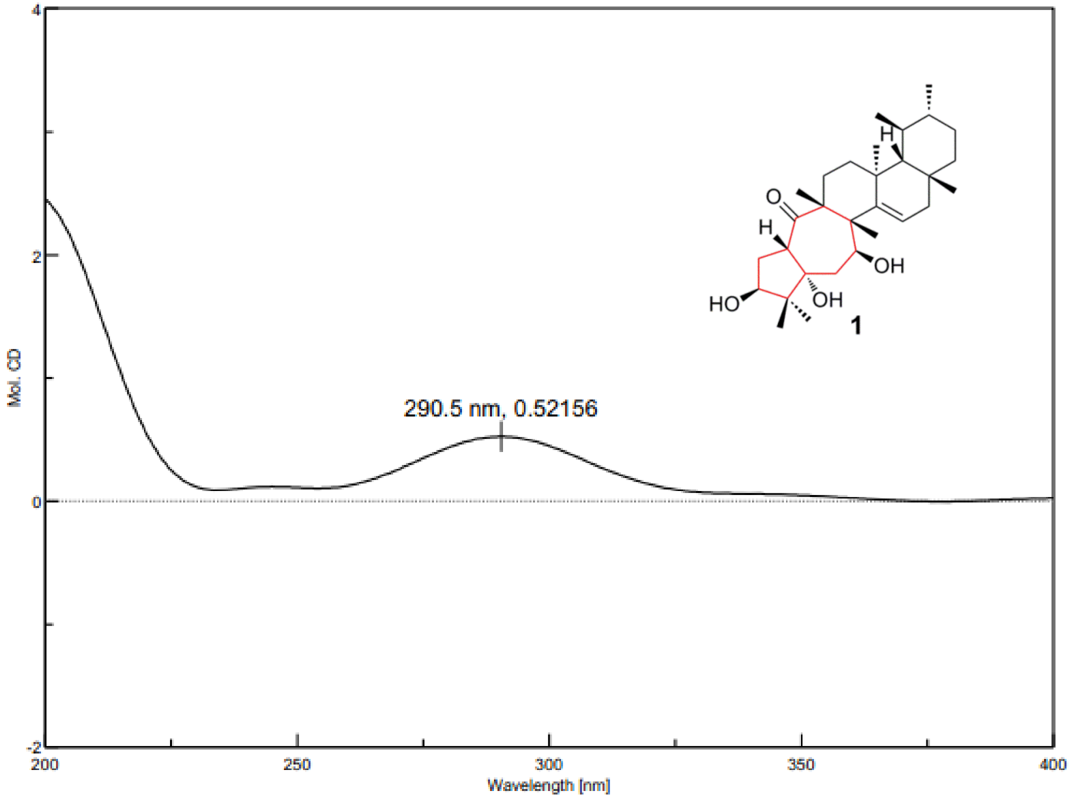

Figure S2. The experimental ECD spectrum of compound **1** in MeOH

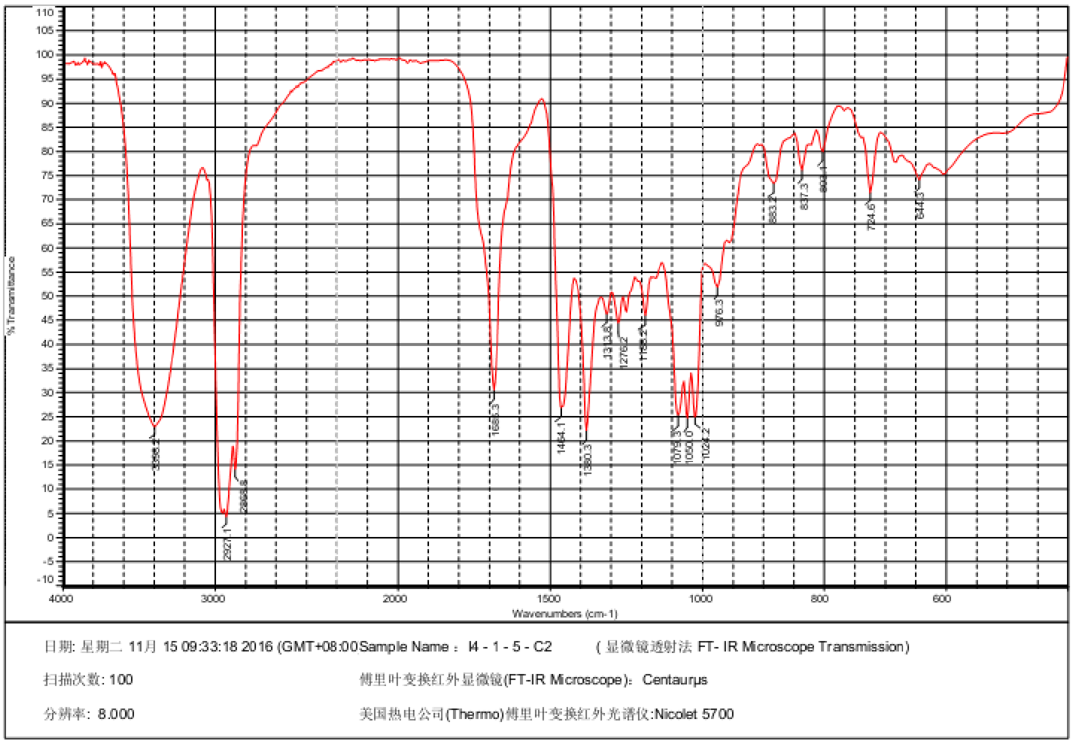

Figure S3. The IR spectrum of compound **1**

MS Formula Results: + Scan (9.072 min) Sub (2016050601.d)

m/z	Ion	Formula	Abundance
495.3438	(M+Na)+	C30 H48 Na O4	60510.5

Best	Formula (M)	Ion Formula	Score	Cross Sco	Mass	Calc Mass	Calc m/z	Diff (ppm)	Abs Diff (ppm)	Mass Match	Abund Match	Spacing Match	DBE
✔	C30 H48 O4	C30 H48 Na O4	98.62		472.3546	472.3553	495.3445	1.41	1.41	99.94	97.76	97.02	7
	C31 H44 N4	C31 H44 N4 Na	97.93		472.3546	472.3566	495.3458	4.23	4.23	99.45	96.29	96.87	12
	C26 H52 N2 O S2	C26 H52 N2 Na O S2	96.7		472.3546	472.3521	495.3413	-5.3	5.3	99.13	97.87	90.43	2
	C25 H49 Cl N4 O2	C25 H49 Cl N4 Na O2	84.46		472.3546	472.3544	495.3436	-0.43	0.43	99.99	54.76	89.04	3

Figure S4. The (+)-HRESIMS data of compound **1**

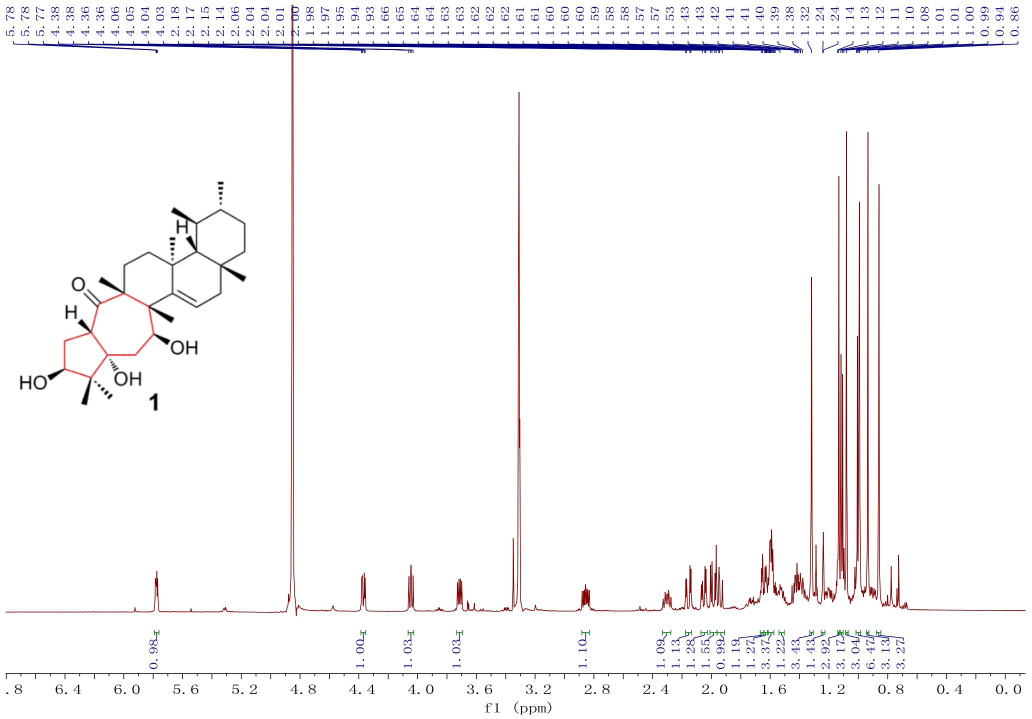

Figure S5. The ^{1}H NMR spectrum of compound **1** in CD_3OD

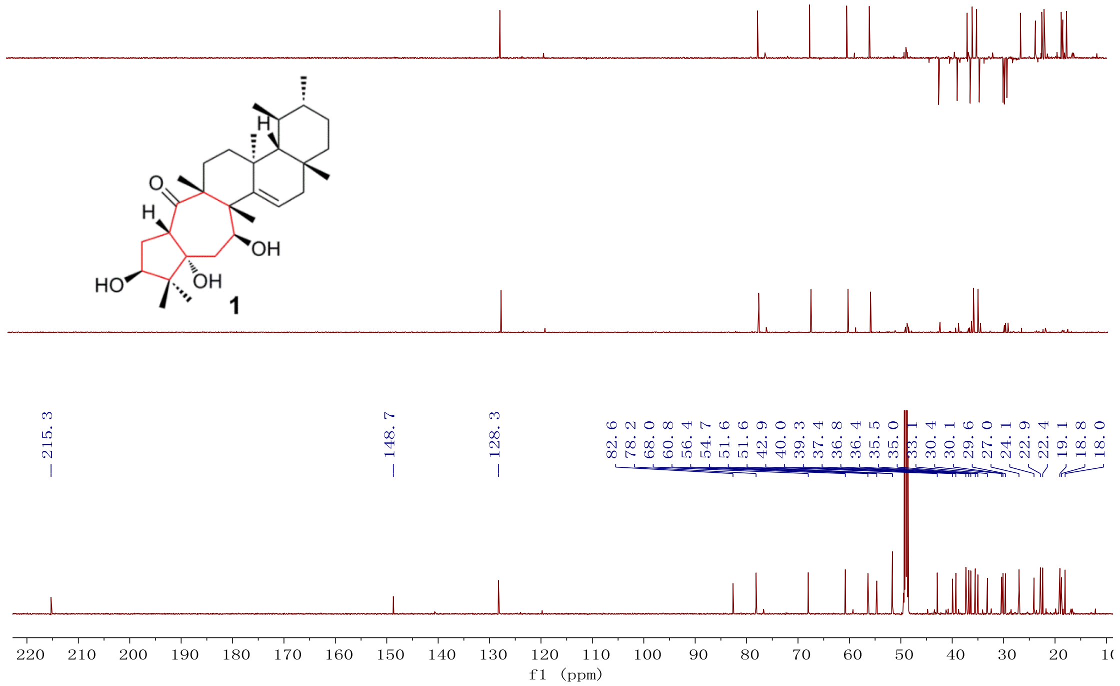

Figure S6. The ^{13}C NMR spectrum and DEPT spectrum of compound **1** in CD_3OD

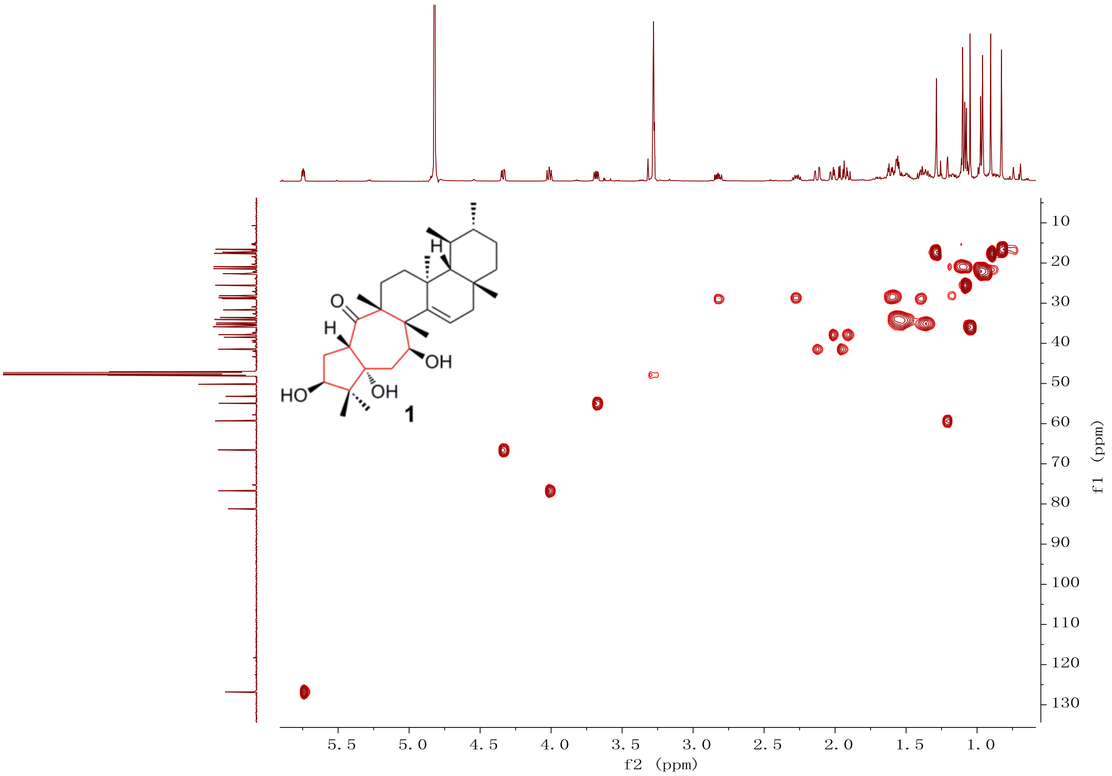

Figure S7. The HSQC spectrum of compound **1** in CD_3OD

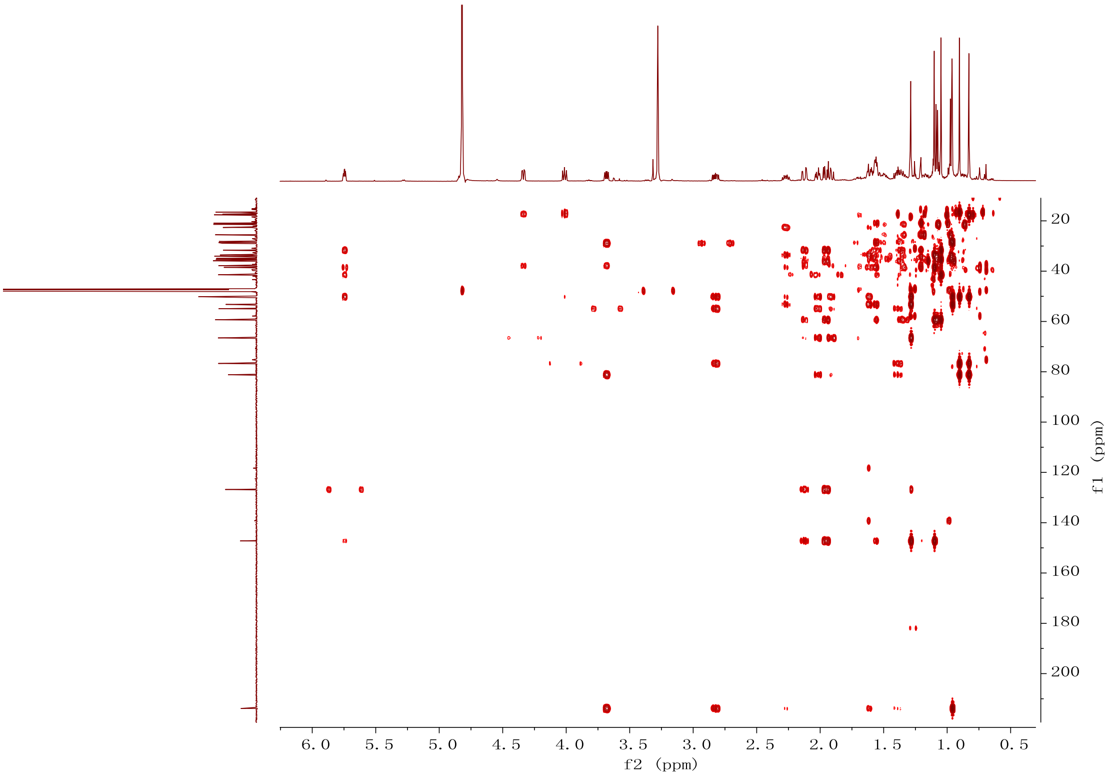

Figure S8. The HMBC spectrum of compound **1** in CD_3OD

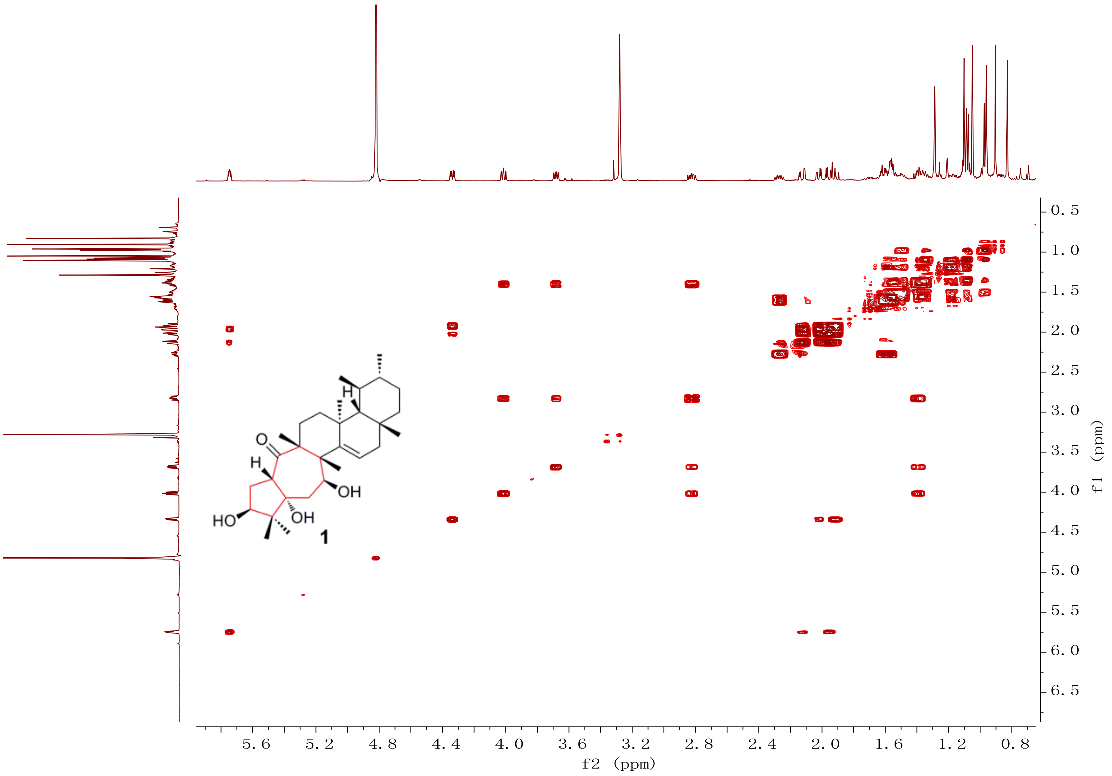

Figure S9. The ^{1}H-^{1}H COSY spectrum of compound **1** in CD_3OD

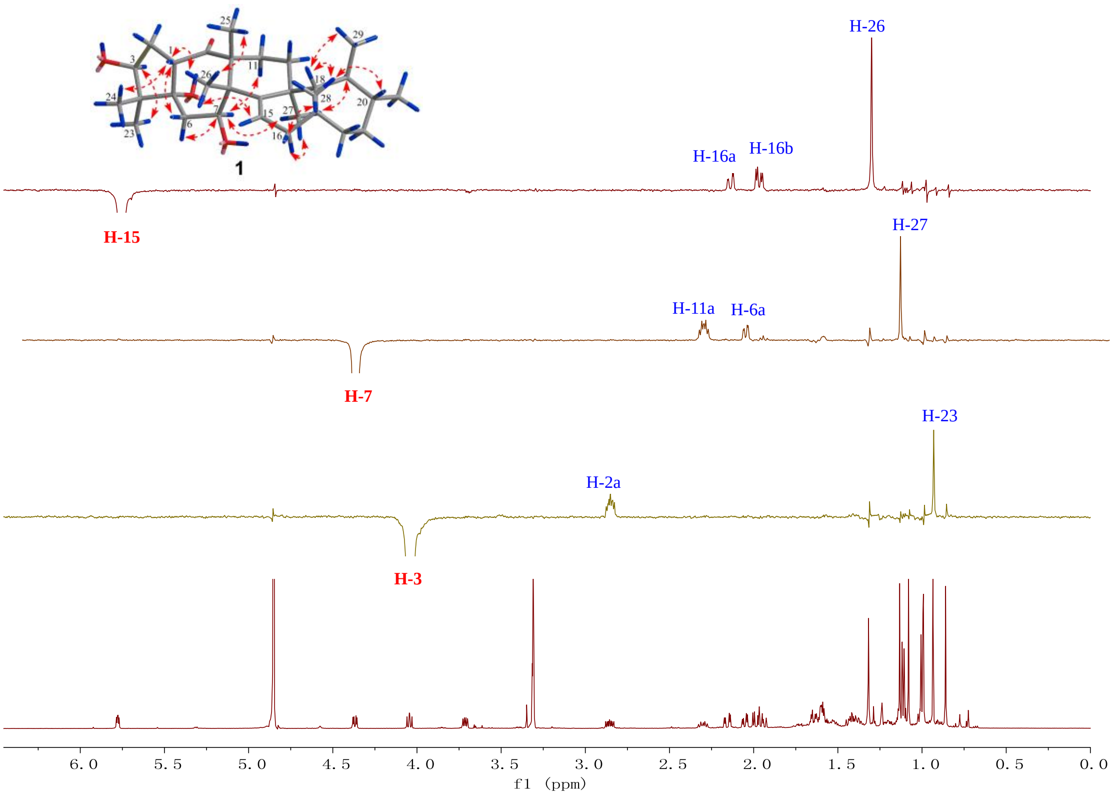

Figure S10. The NOE spectrum of compound **1** in CD_3OD

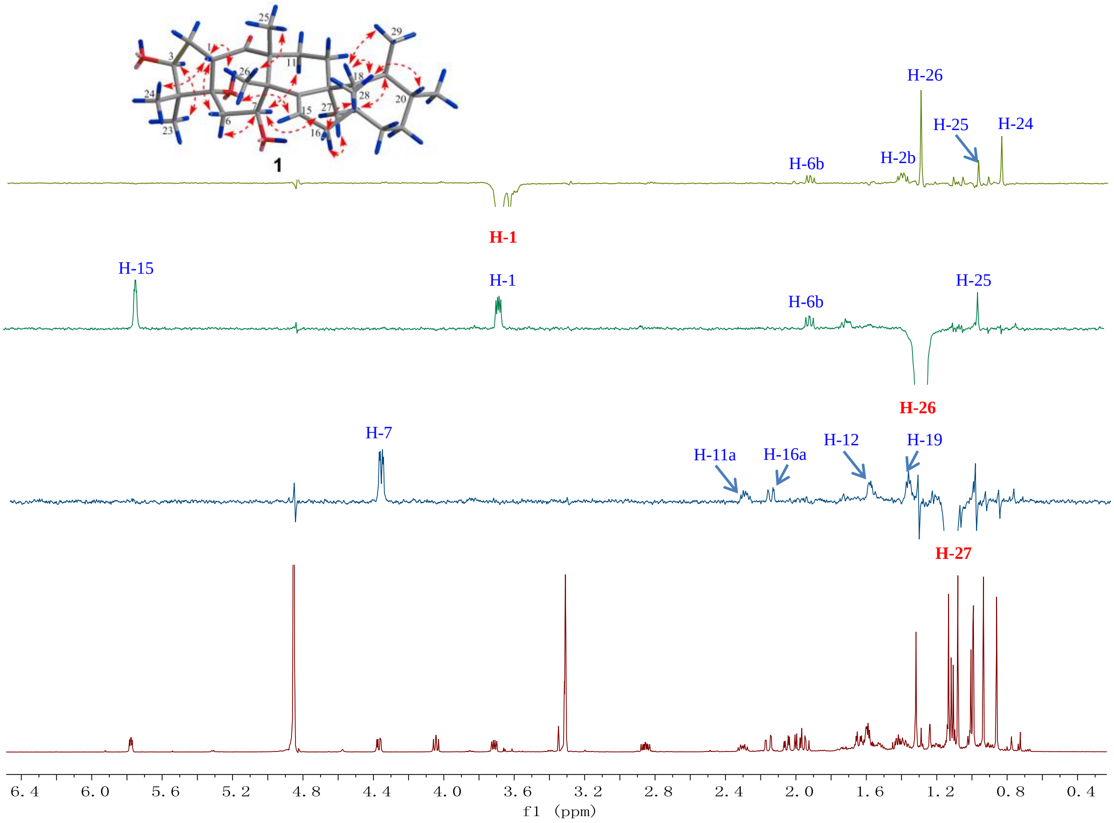

Figure S10. The NOE spectrum of compound **1** in CD_3OD

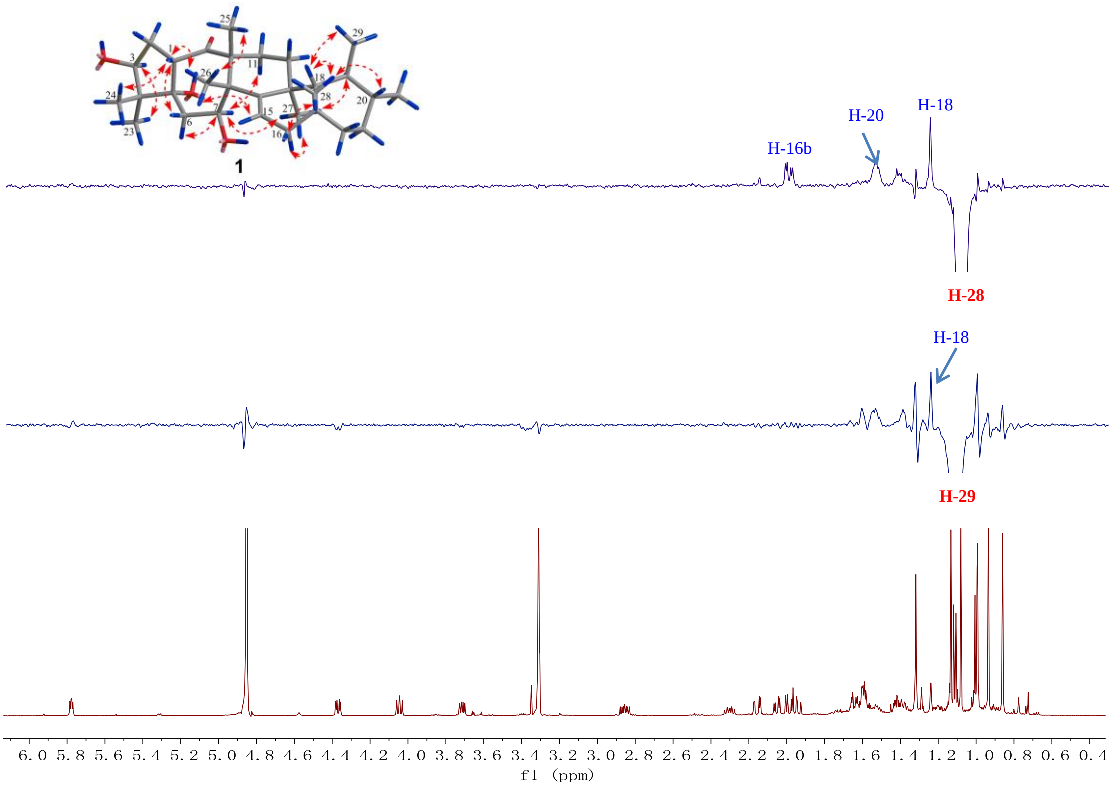

Figure S10. The NOE spectrum of compound **1** in CD_3OD

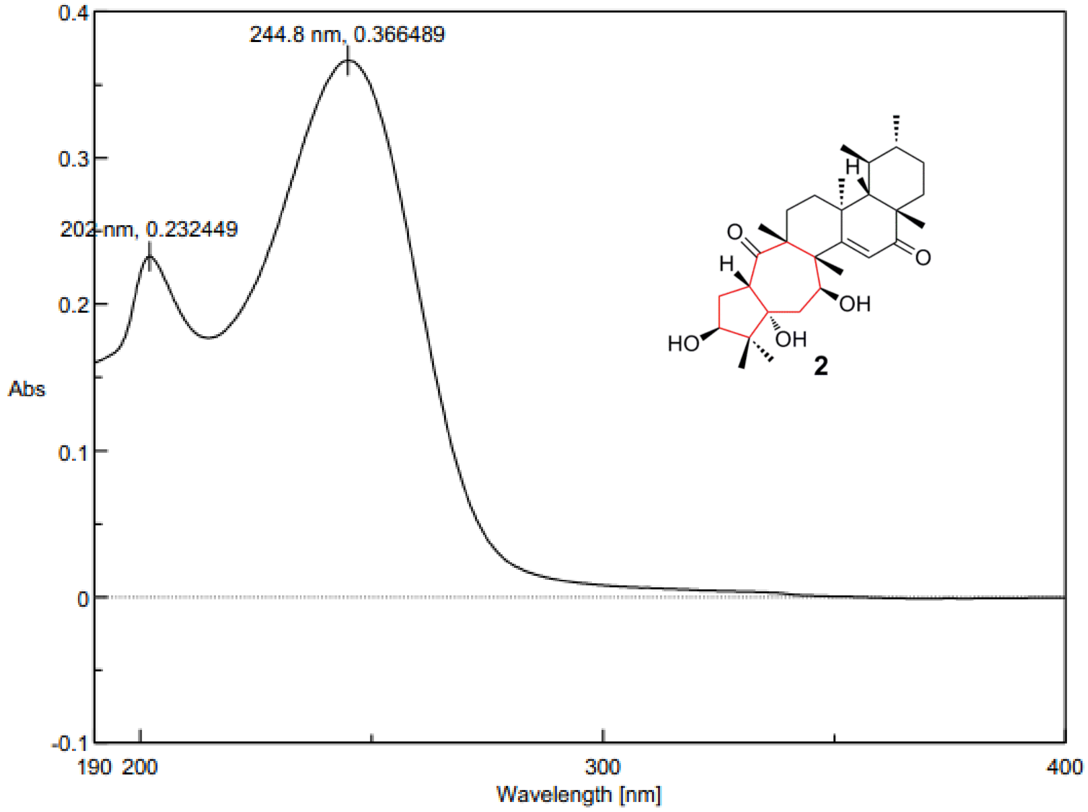

Figure S11. The UV spectrum of compound **2** in MeOH

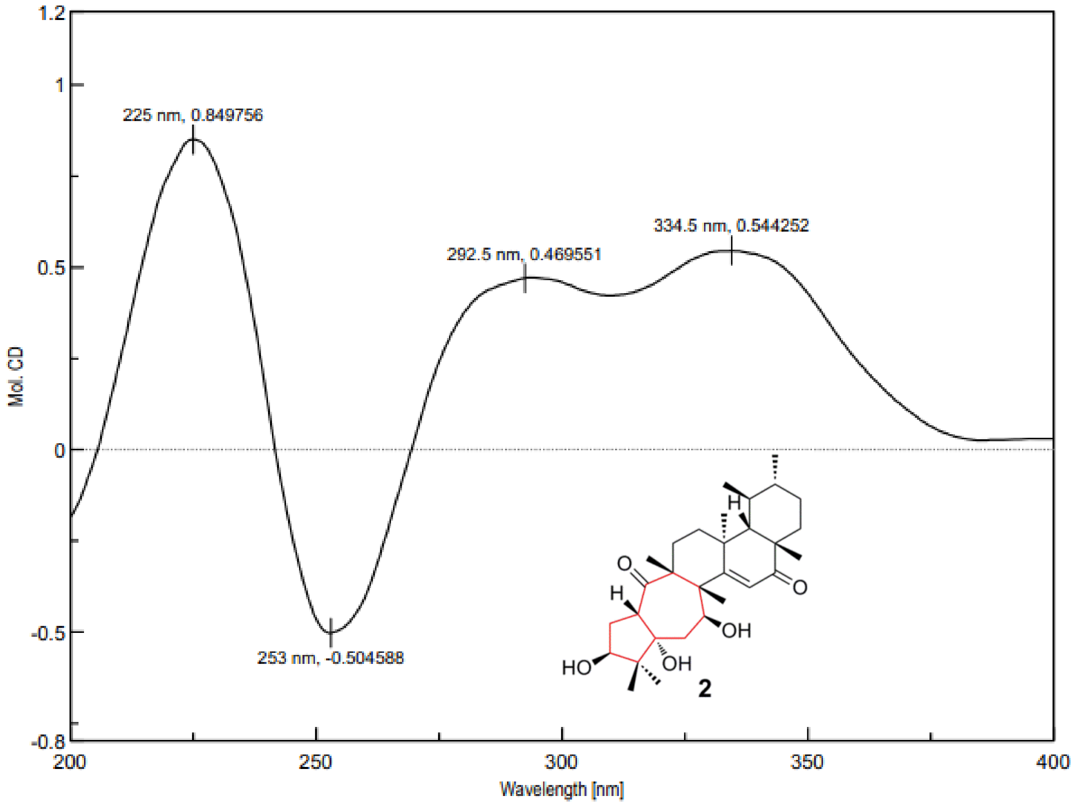

Figure S12. The experimental ECD spectrum of compound **2** in MeOH

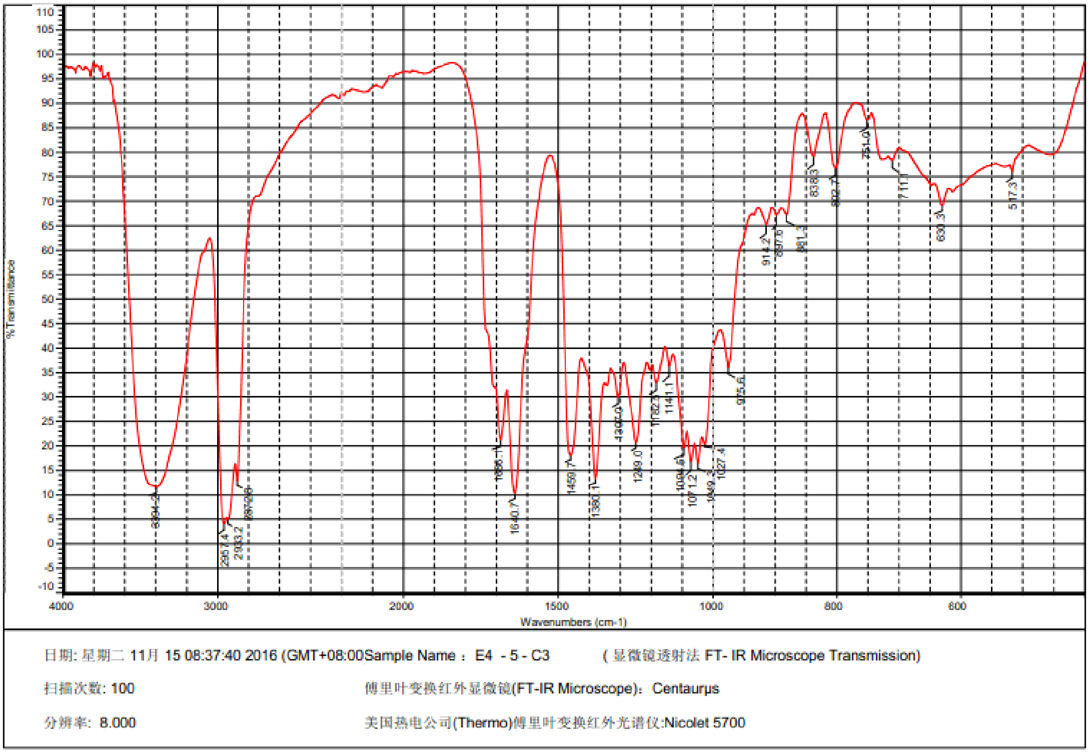

Figure S13. The IR spectrum of compound **2**

MS Formula Results: + Scan (7.953 min) Sub (2016110306.d)

m/z	Ion	Formula	Abundance
487.3428	(M+H)+	C30 H47 O5	557300.4

Best	Formula (M)	Ion Formula	Score	Cross Sco	Mass	Calc Mass	Calc m/z	Diff (ppm)	Abs Diff (ppm)	Mass Match	Abund Match	Spacing Match	DBE
☑	C30 H46 O5	C30 H47 O5	99.89		486.3355	486.3345	487.3418	-2.07	2.07	99.85	99.87	99.99	8
☐	C27 H50 O5 S	C27 H51 O5 S	98.93		486.3355	486.3379	487.3452	4.85	4.85	99.2	98.02	99.5	3
☐	C31 H50 S2	C31 H51 S2	97.86		486.3355	486.3354	487.3427	-0.3	0.3	100	93.22	99.16	7

Figure S14. The (+)-HRESIMS data of compound **2**

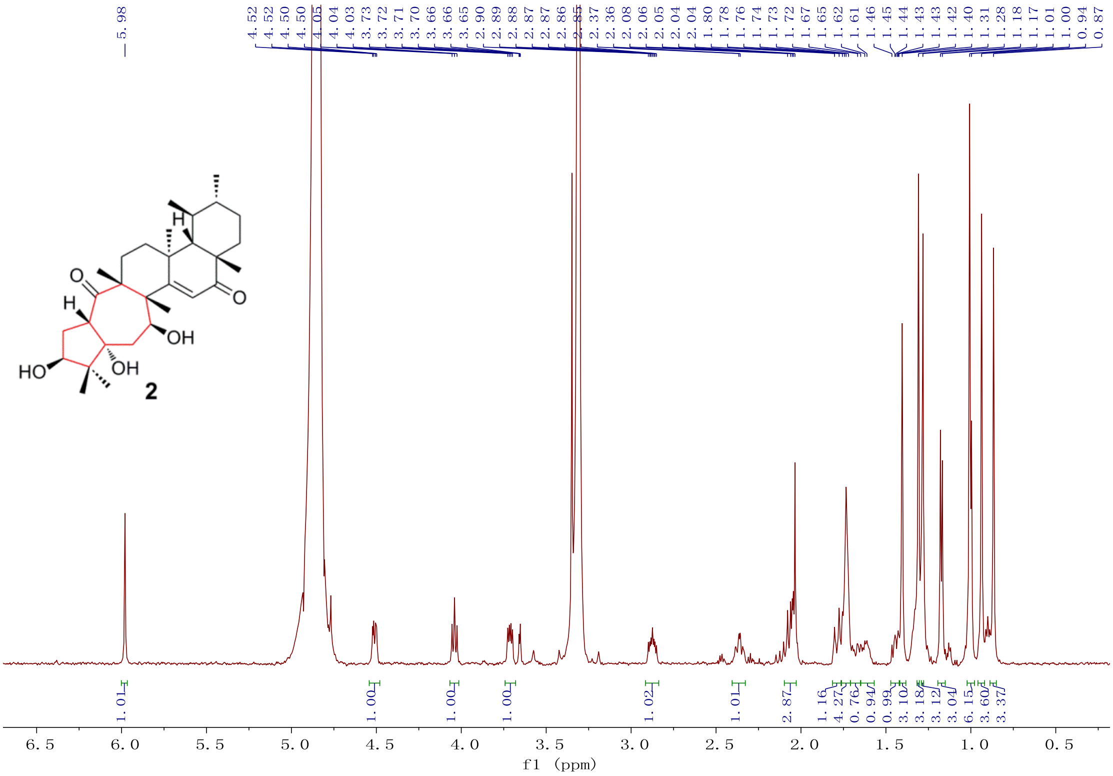

Figure S15. The ^{1}H NMR spectrum of compound **2** in CD_3OD

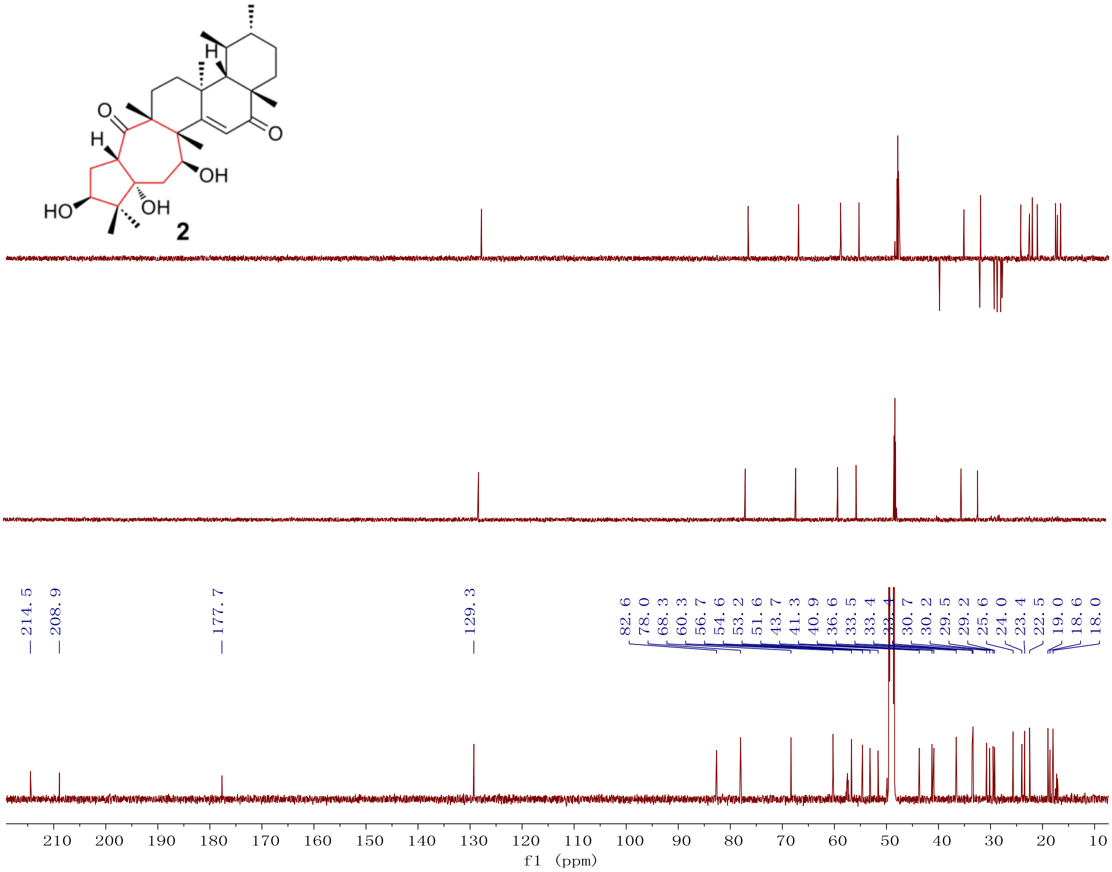

Figure S16. The ^{13}C NMR spectrum and DEPT spectrum of compound **2** in CD_3OD

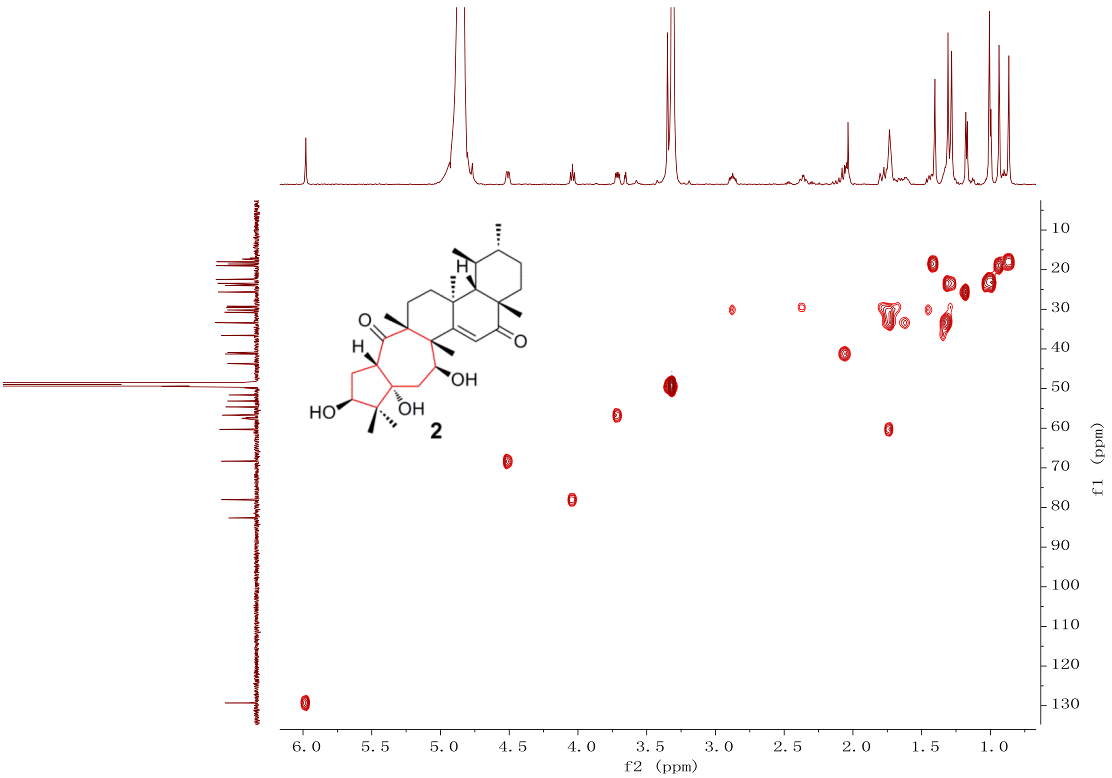

Figure S17. The HSQC spectrum of compound **2** in CD_3OD

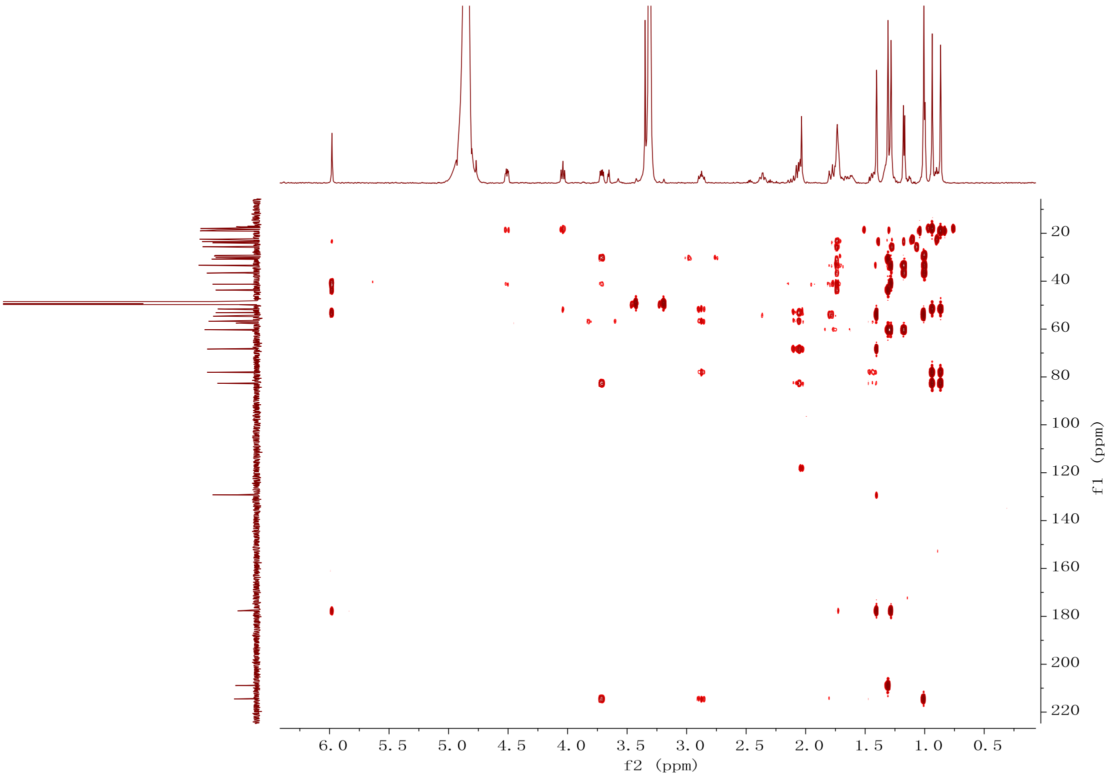

Figure S18. The HMBC spectrum of compound **2** in CD_3OD

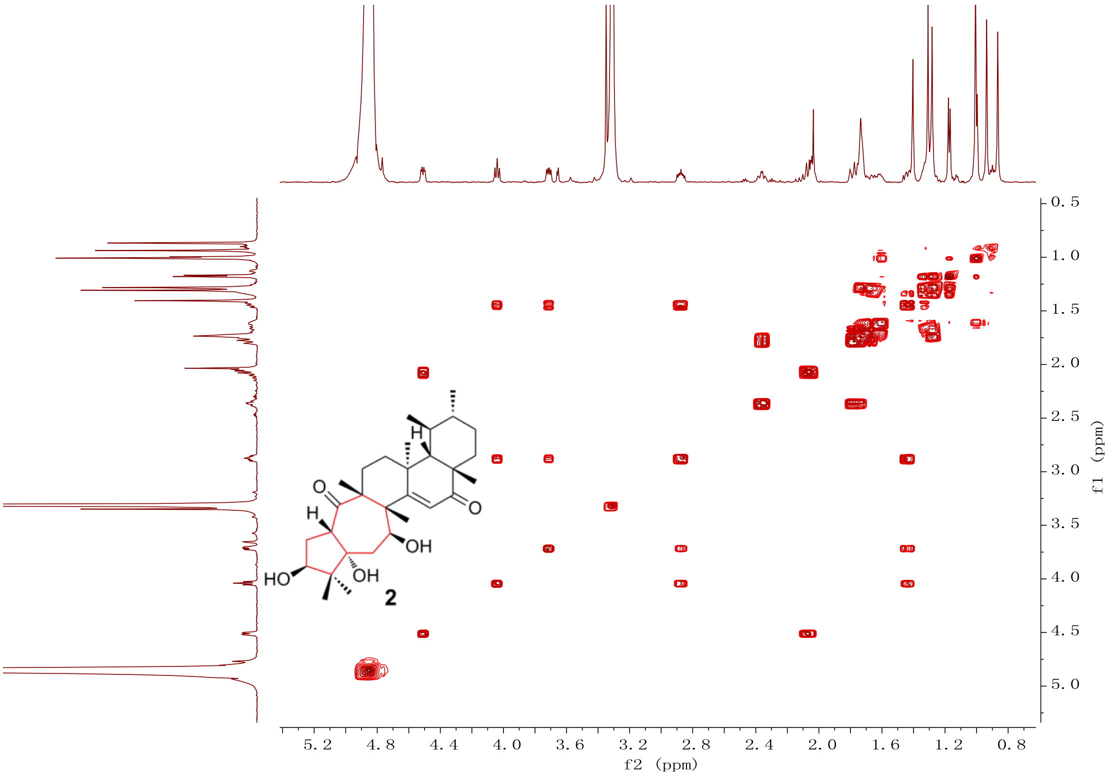

Figure S19. The ^{1}H-^{1}H COSY spectrum of compound **2** in CD_3OD

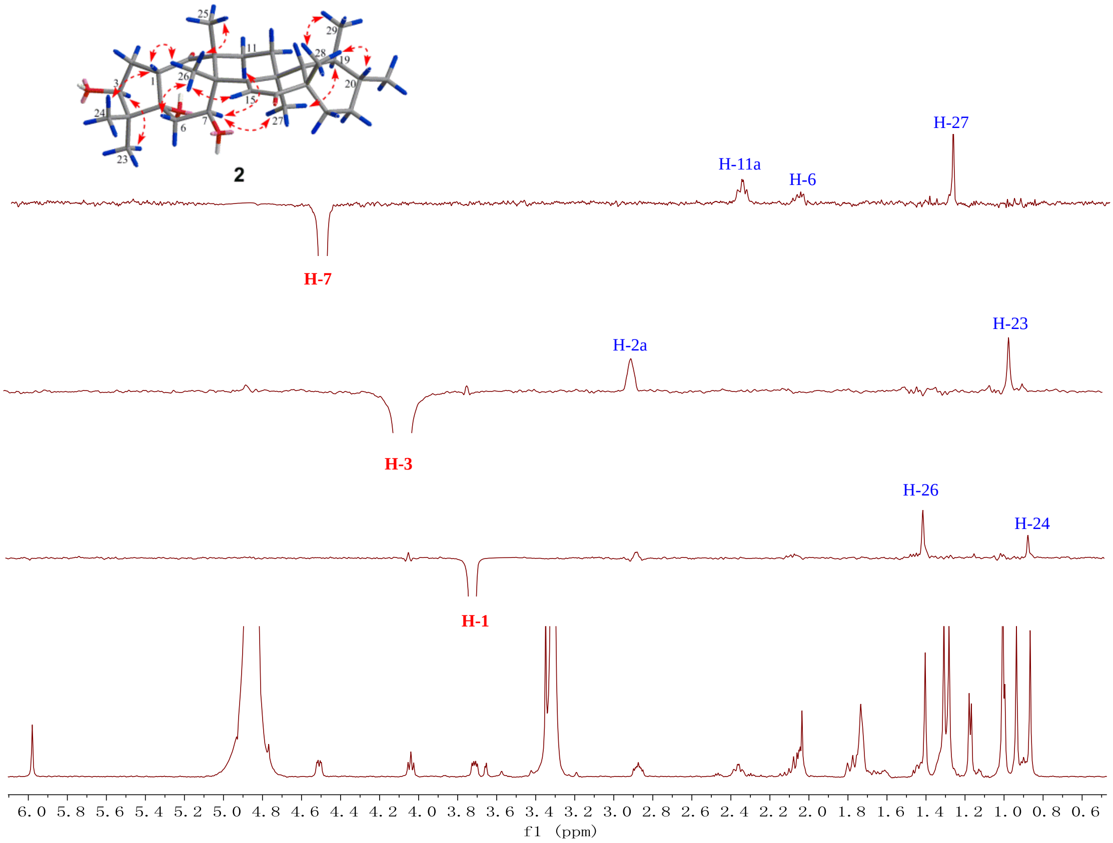

Figure S20. The NOE spectrum of compound **2** in CD_3OD

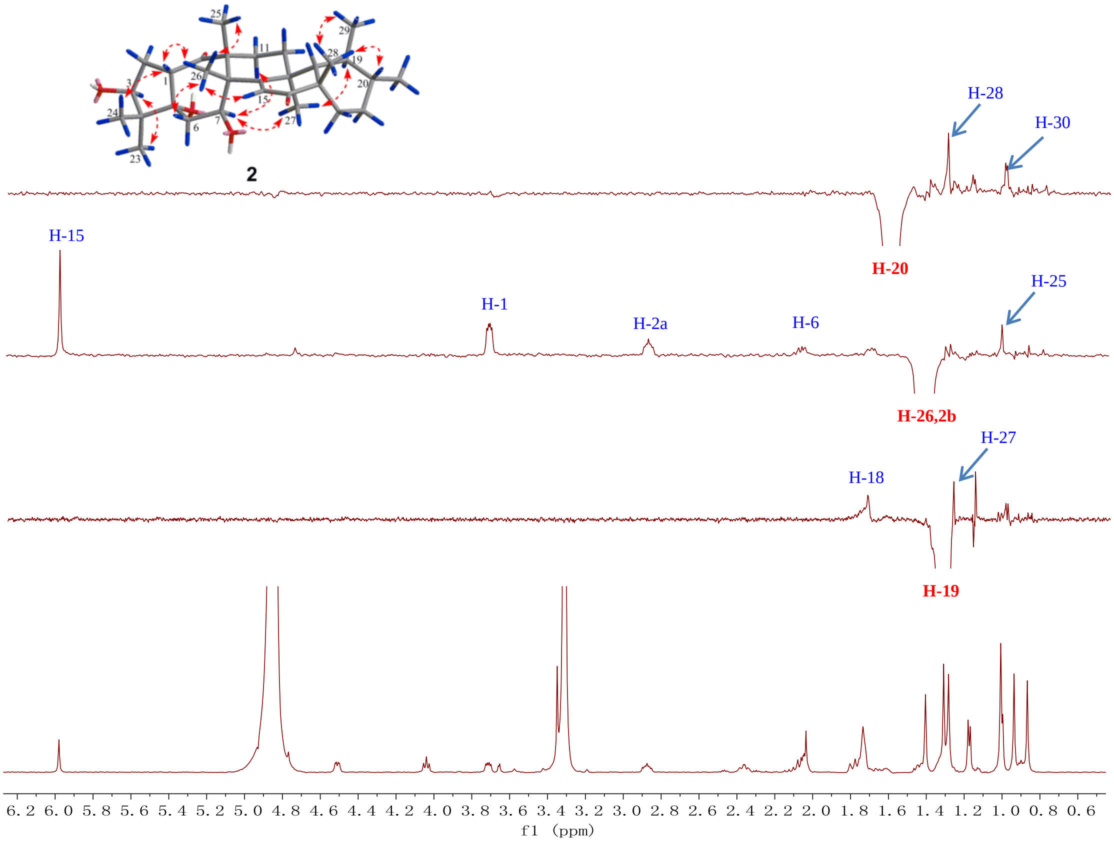

Figure S20. The NOE spectrum of compound **2** in CD_3OD

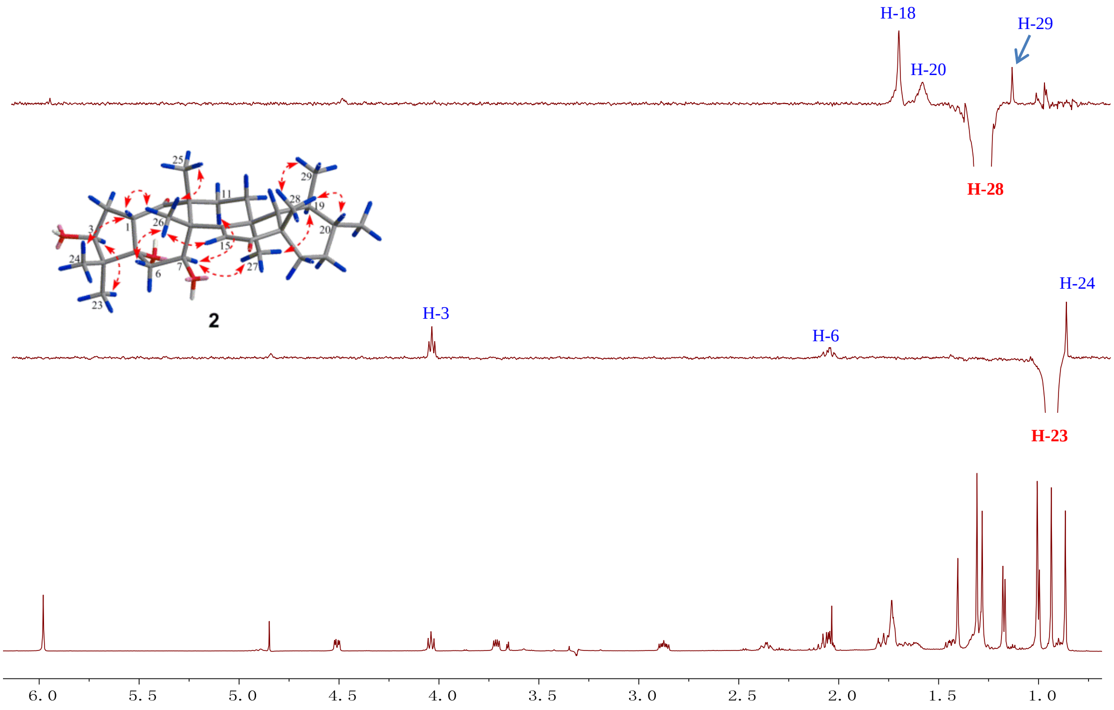

Figure S20. The NOE spectrum of compound **2** in CD_3OD

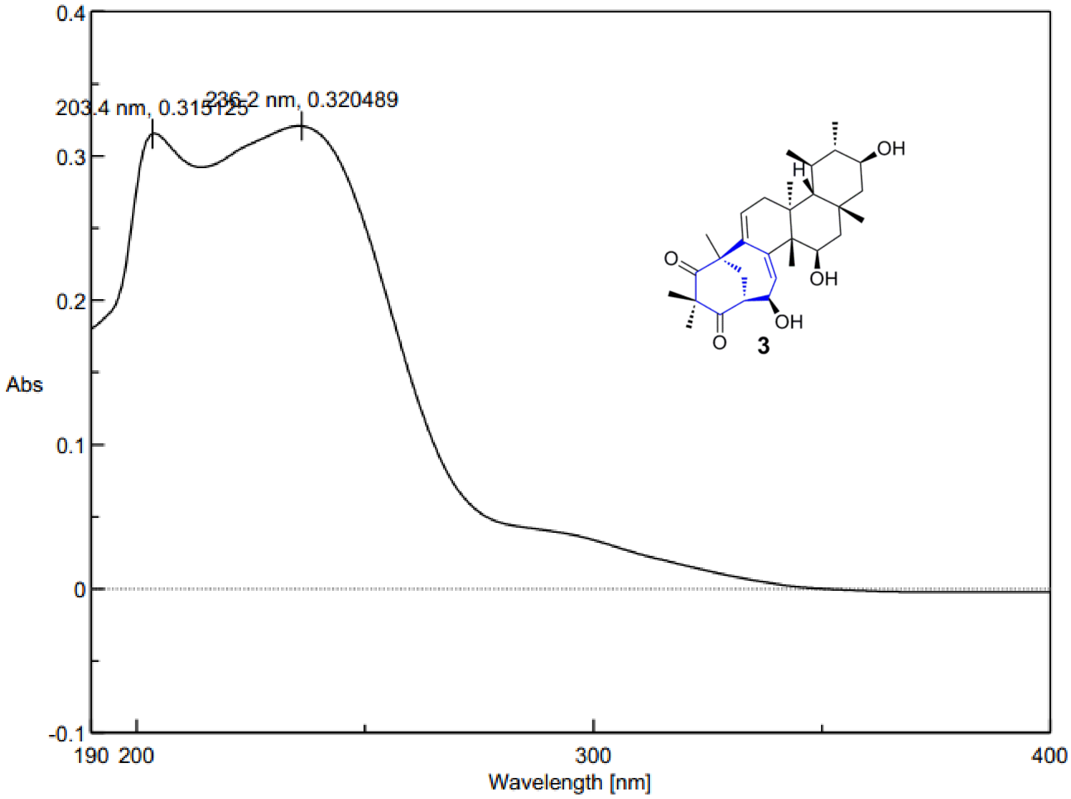

Figure S21. The UV spectrum of compound **3** in MeOH

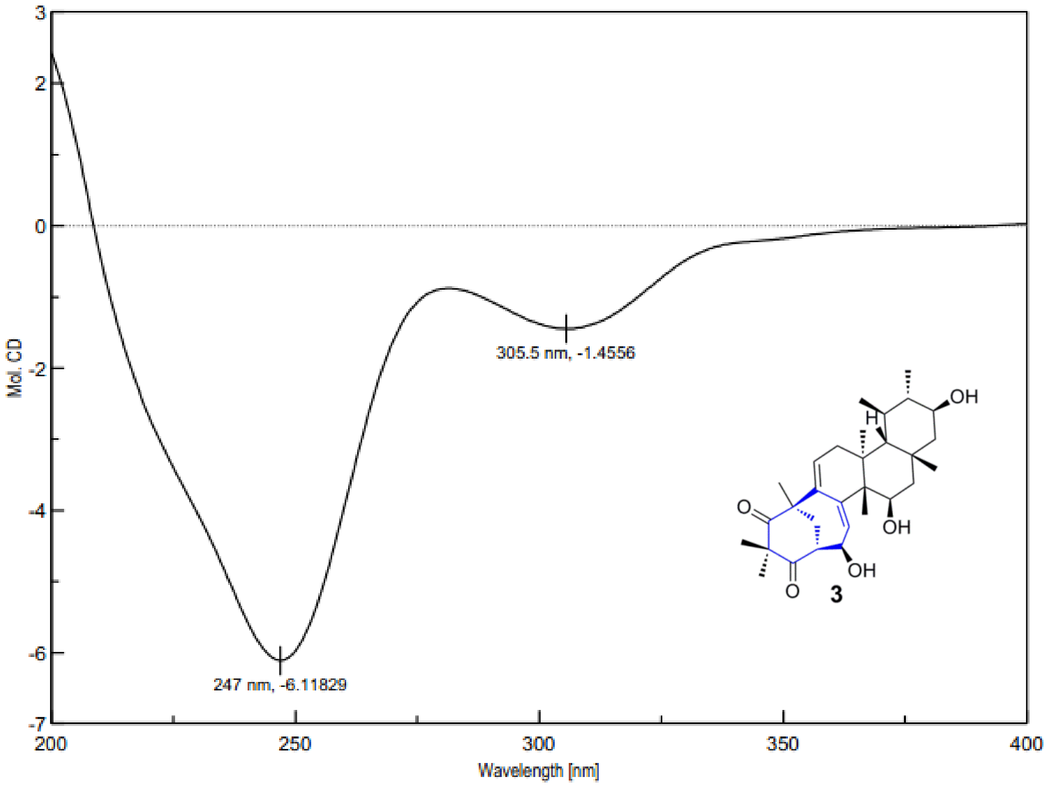

Figure S22. The experimental ECD spectrum of compound **3** in MeOH

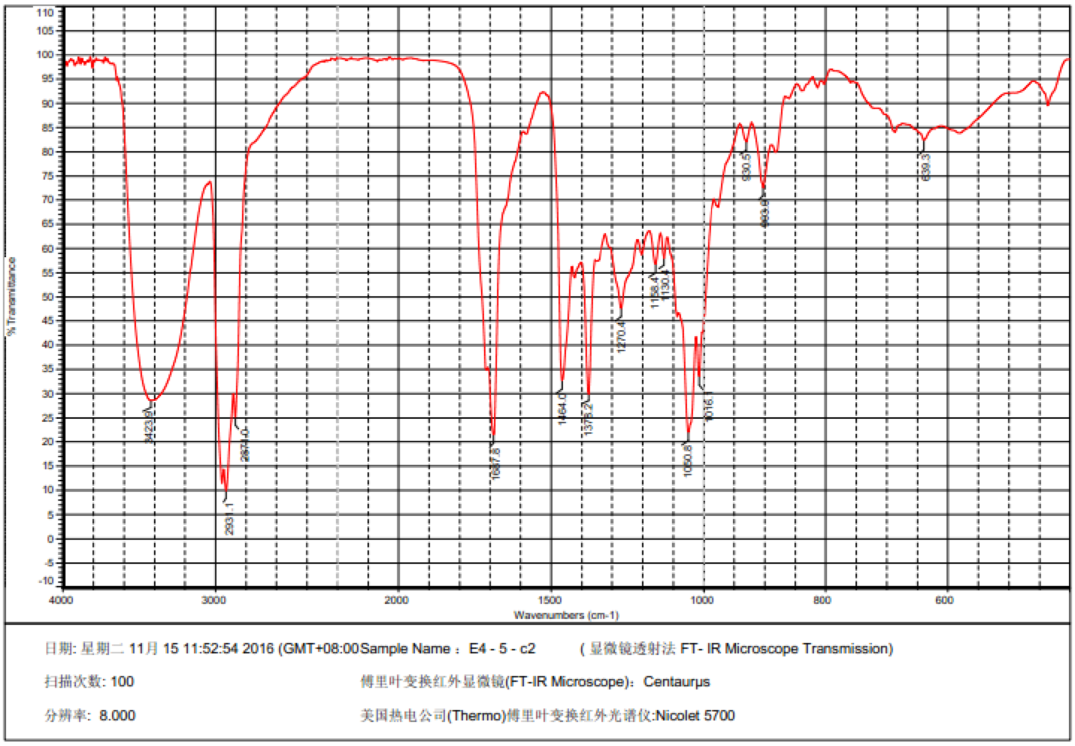

Figure S23. The IR spectrum of compound **3**

MS Formula Results: + Scan (7.916 min) Sub (2016110307.d)

m/z	Ion	Formula	Abundance
507.3081	(M+Na)+	C30 H44 Na O5	206581.3

Best	Formula (M)	Ion Formula	Score	Cross Sco	Mass	Calc Mass	Calc m/z	Diff (ppm)	Abs Diff (ppm)	Mass Match	Abund Match	Spacing Match	DBE
✓	C30 H44 O5	C30 H44 Na O5	99.82		484.3189	484.3189	507.3081	-0.01	0.01	100	99.47	99.91	9
	C34 H44 S	C34 H44 Na S	98.88		484.3189	484.3164	507.3056	-5.19	5.19	99.16	98.25	99.08	13
	C22 H48 N2 O7 S	C22 H48 N2 Na O7 S	98.36		484.3189	484.3182	507.3074	-1.39	1.39	99.94	95.61	98.5	0
	C31 H48 S2	C31 H48 Na S2	98.27		484.3189	484.3197	507.309	1.76	1.76	99.9	95.62	98.18	8

Figure S24. The (+)-HRESIMS data of compound **3**

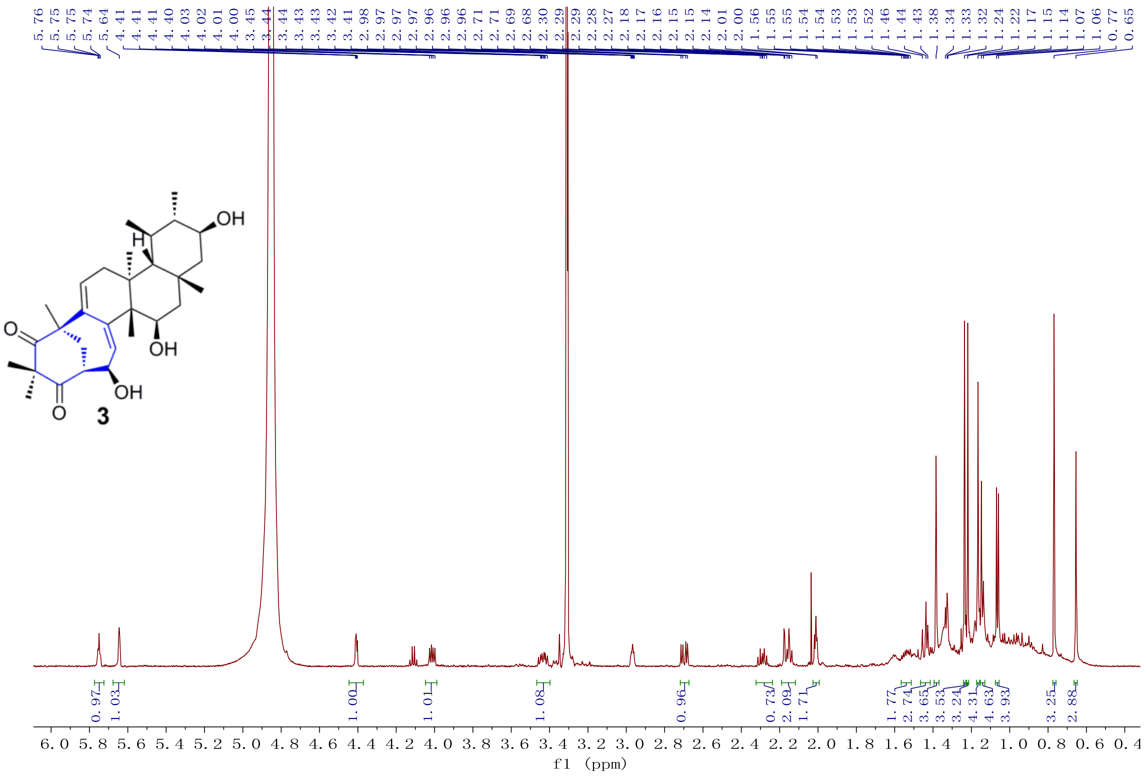

Figure S25. The ^{1}H NMR spectrum of compound **3** in CD_3OD

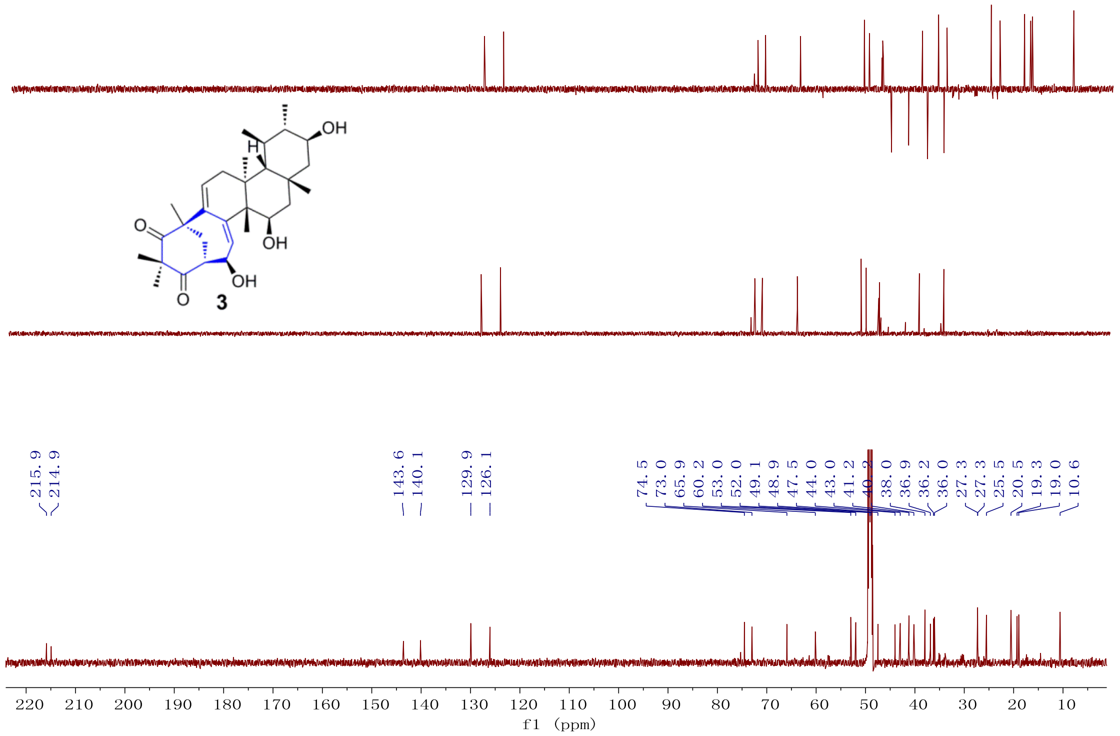

Figure S26. The ^{13}C NMR spectrum and DEPT spectrum of compound **3** in CD_3OD

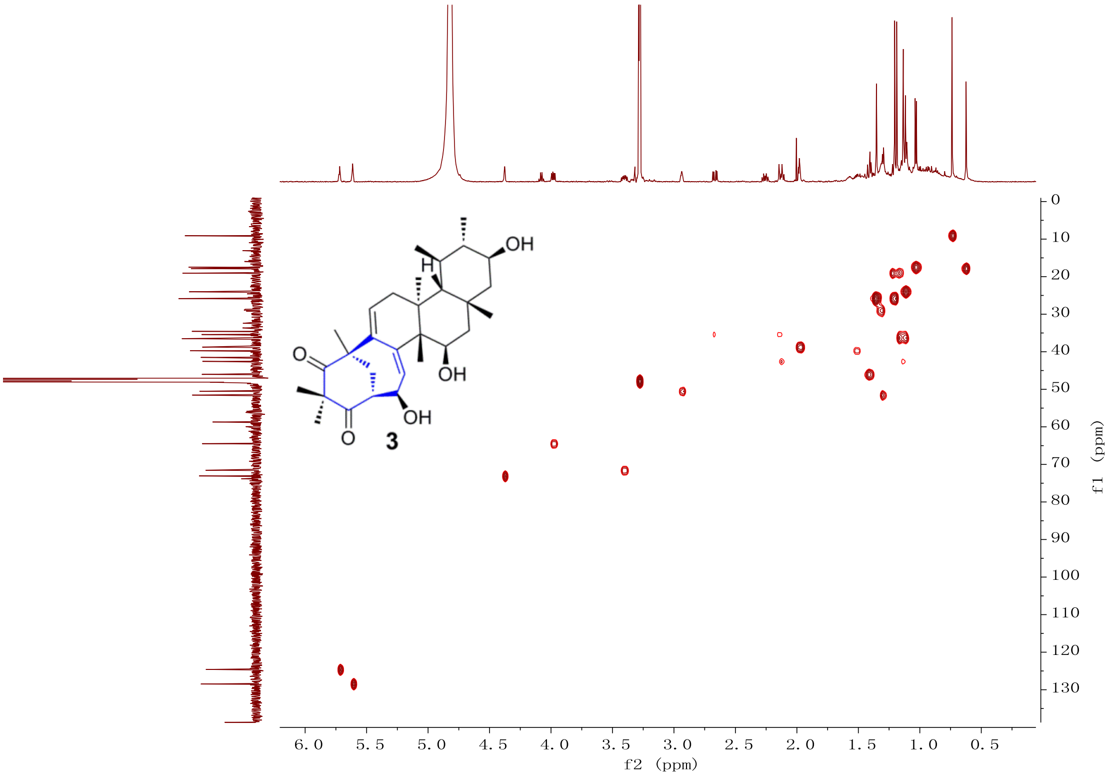

Figure S27. The HSQC spectrum of compound **3** in CD_3OD

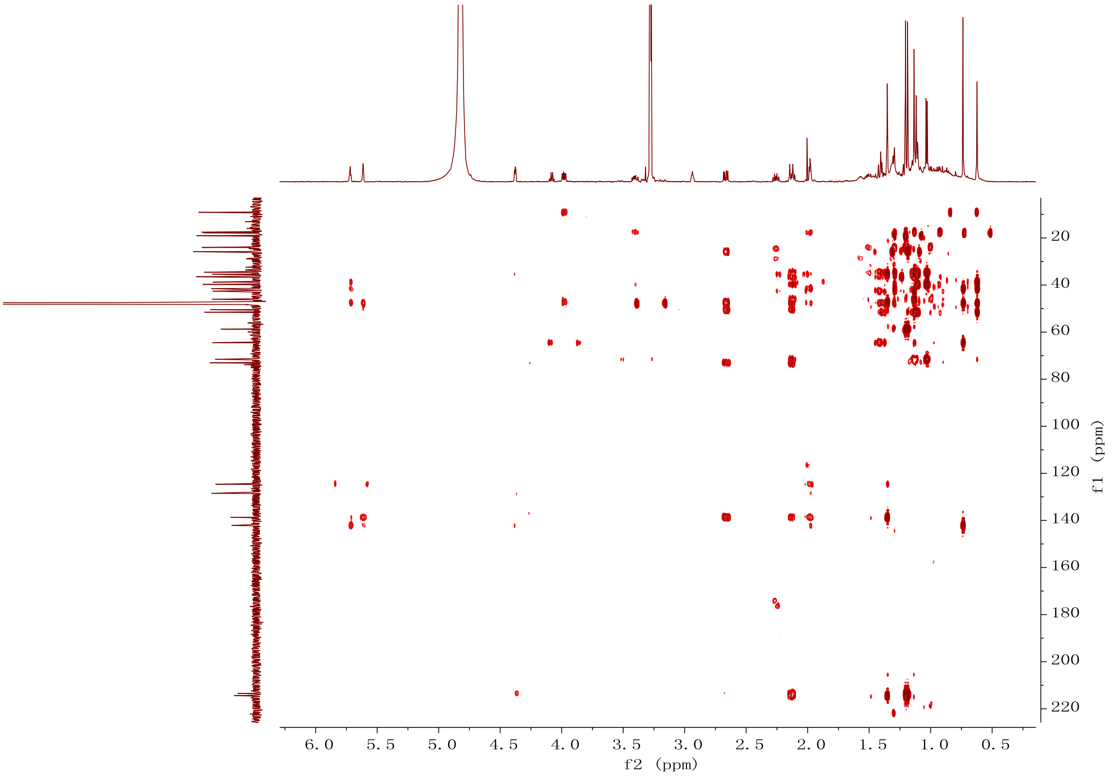

Figure S28. The HMBC spectrum of compound **3** in CD_3OD

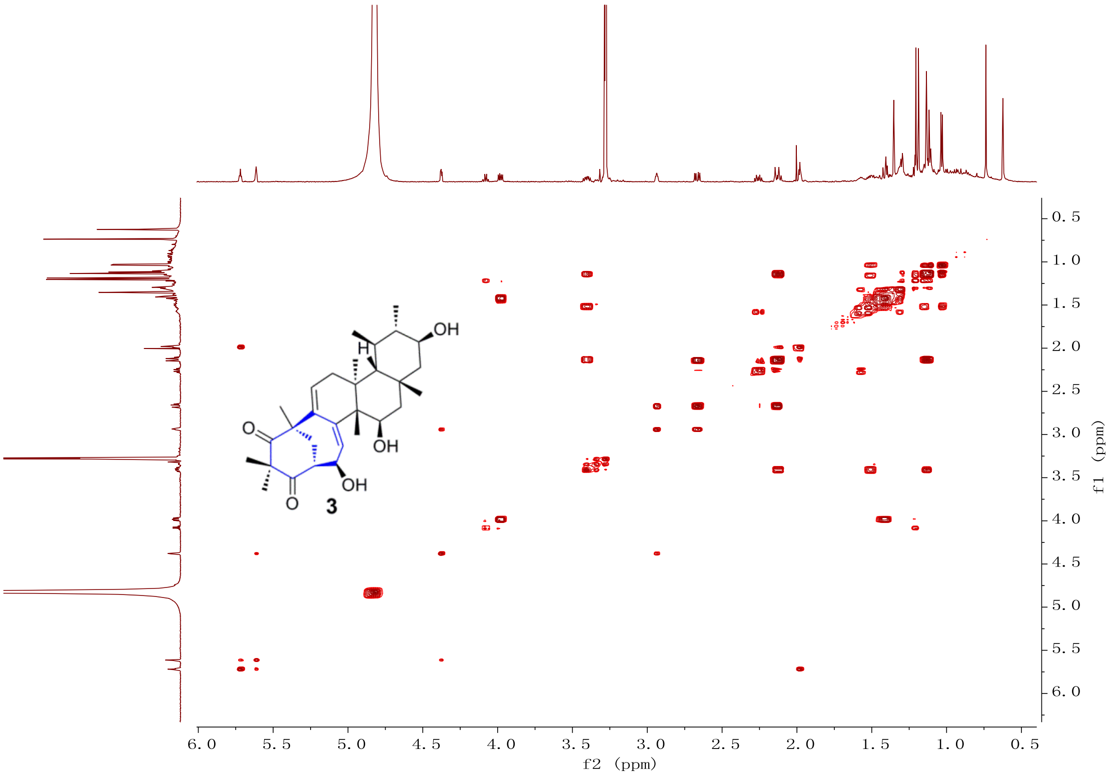

Figure S29. The ^{1}H-^{1}H COSY spectrum of compound **3** in CD_3OD

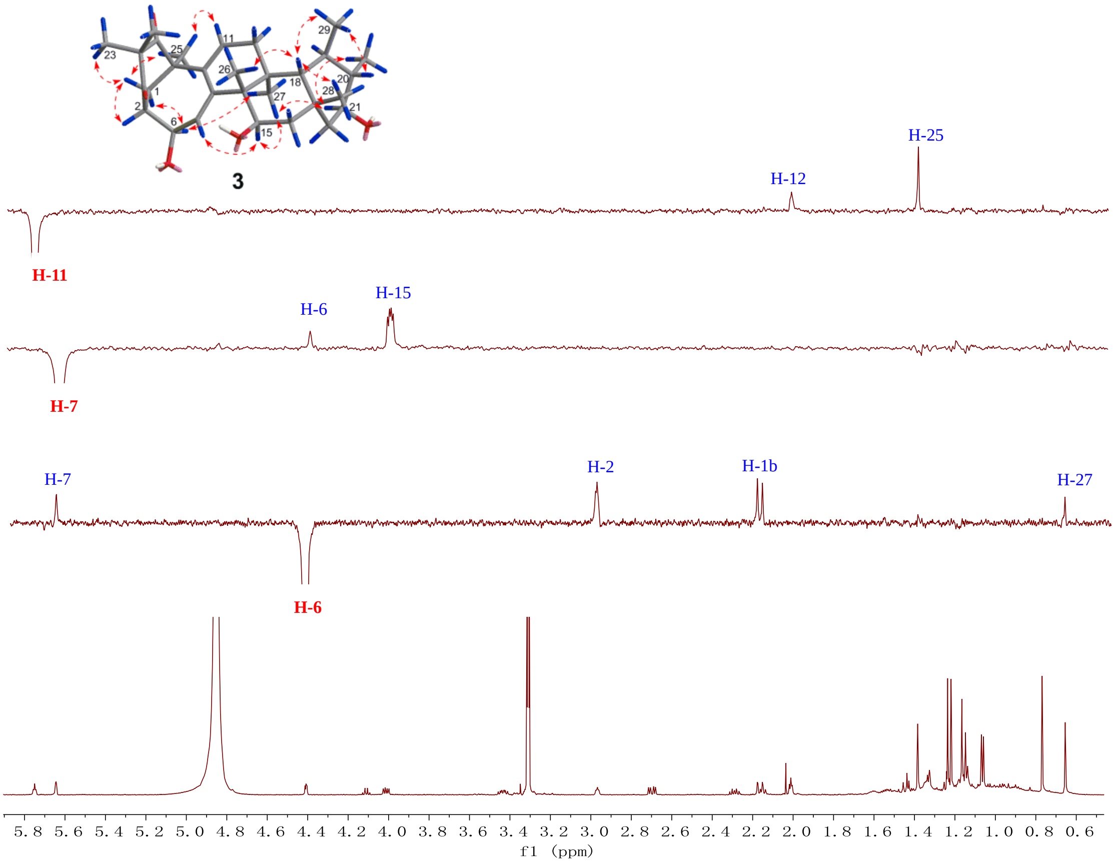

Figure S30. The NOE spectrum of compound **3** in CD_3OD

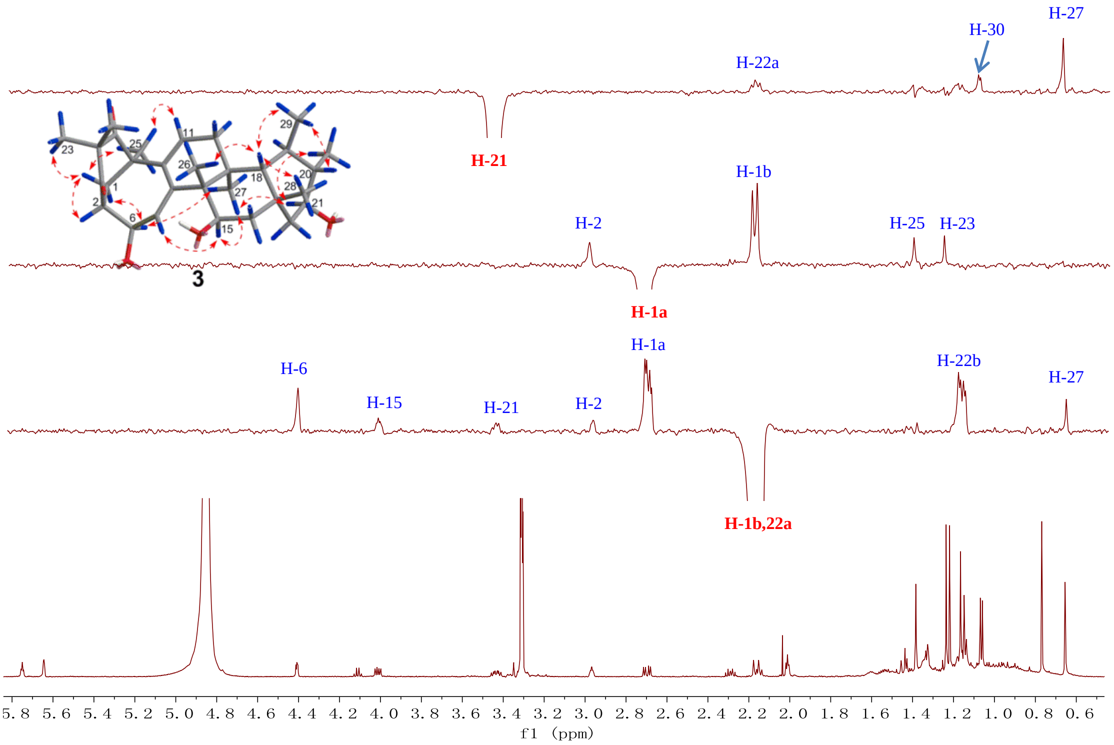

Figure S30. The NOE spectrum of compound **3** in CD_3OD

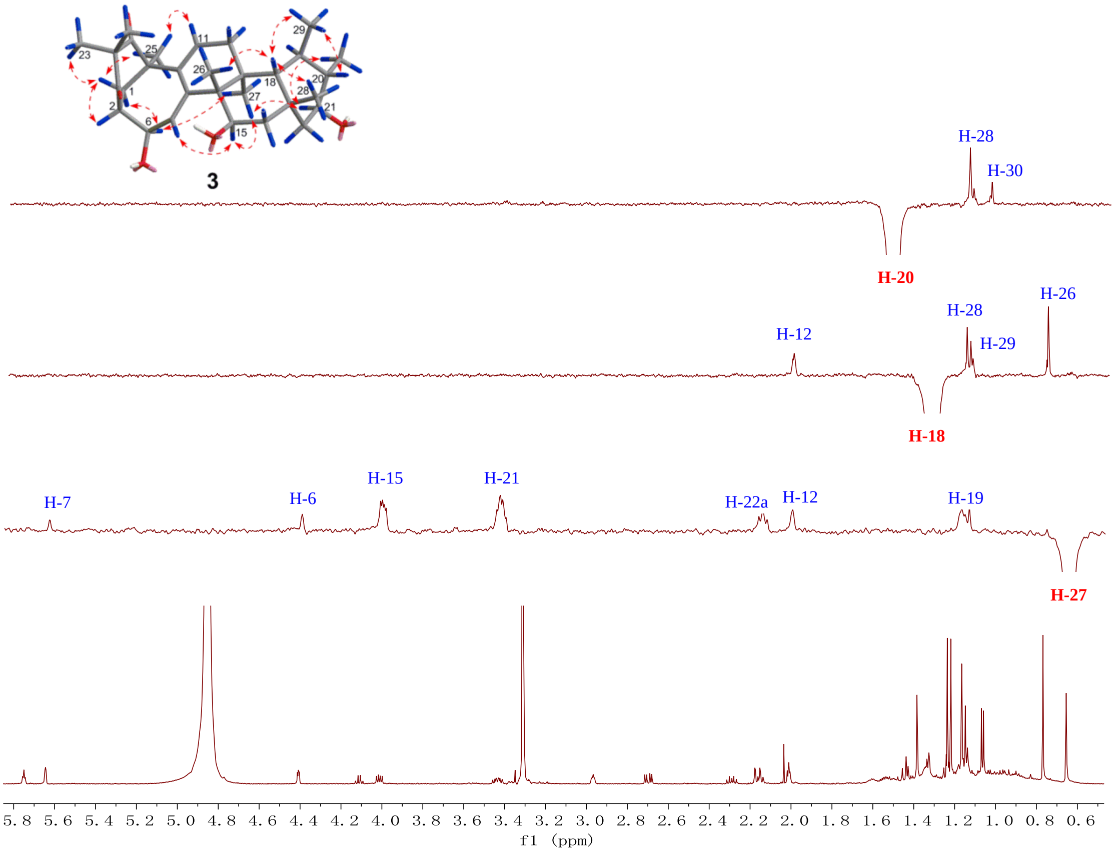

Figure S30. The NOE spectrum of compound **3** in CD_3OD

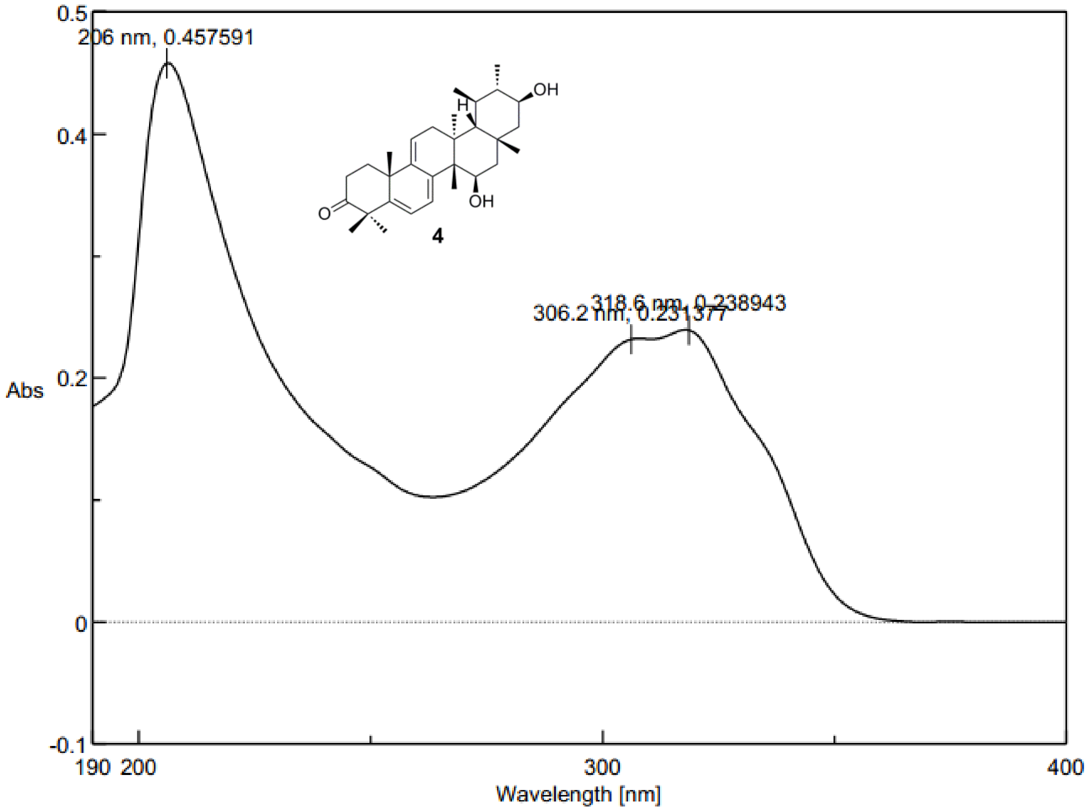

Figure S31. The UV spectrum of compound **4** in MeOH

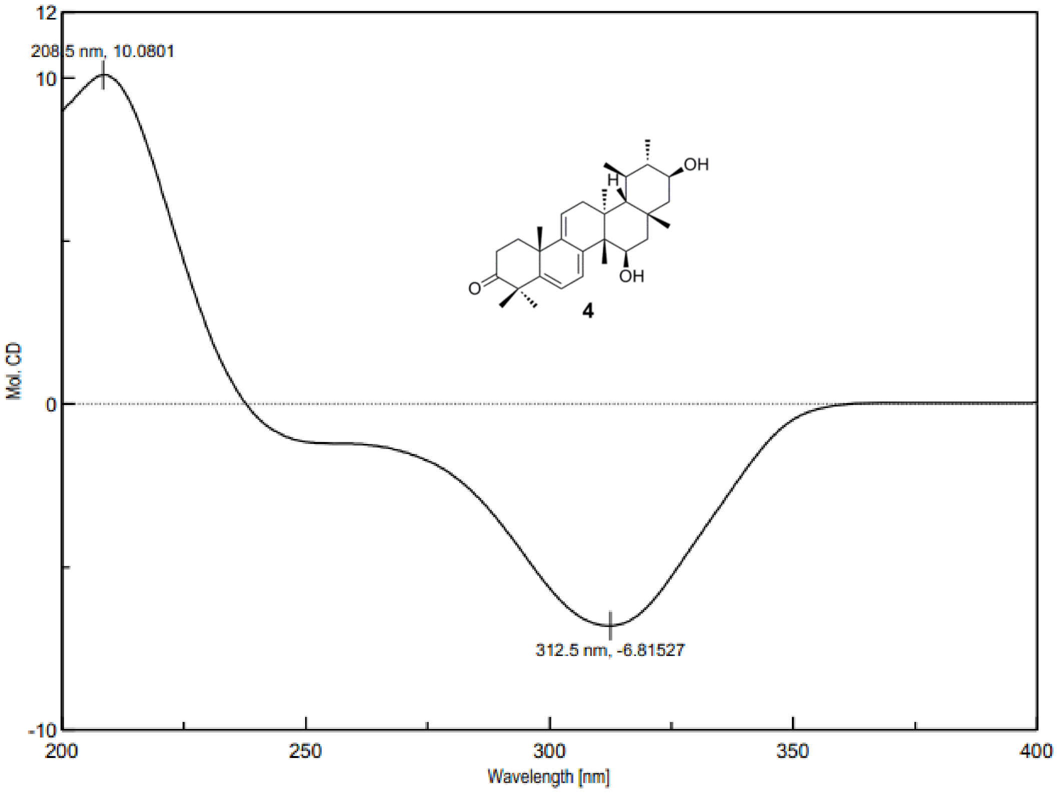

Figure S32. The experimental ECD spectrum of compound **4** in MeOH

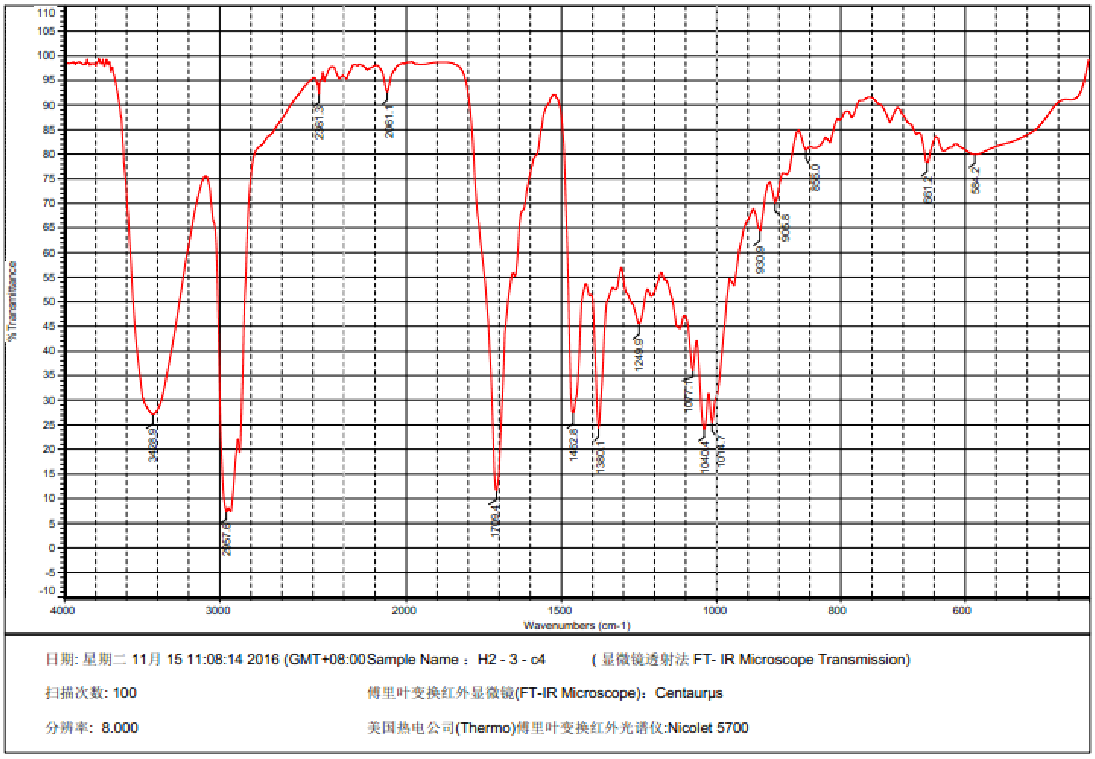

Figure S33. The IR spectrum of compound **4**

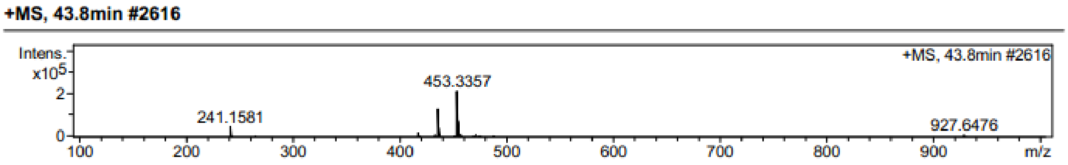

Meas. m/z	#	Formula	m/z	err [mDa]	err [ppm]	mSigma	rdb	N-Rule	e⁻ Conf
453.3357	1	C 30 H 45 O 3	453.3363	0.6	1.3	16.7	8.5	ok	even

Figure S34. The (+)-HRESIMS data of compound **4**

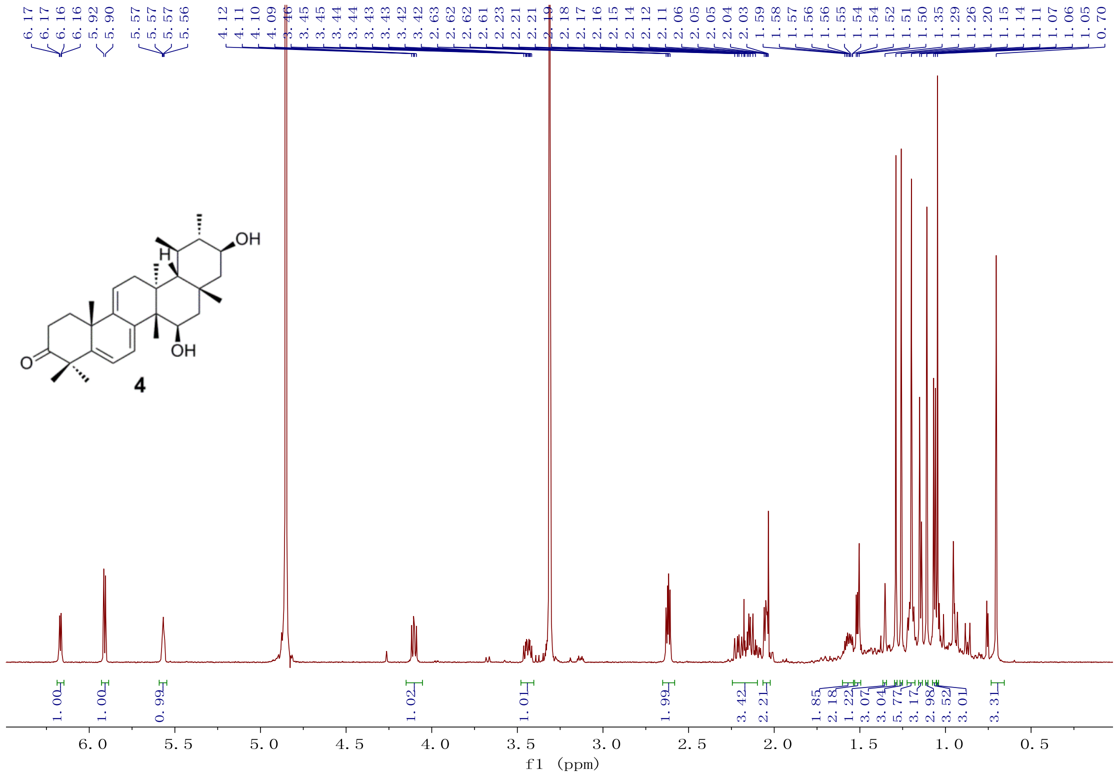

Figure S35. The ^{1}H NMR spectrum of compound **4** in CD_3OD

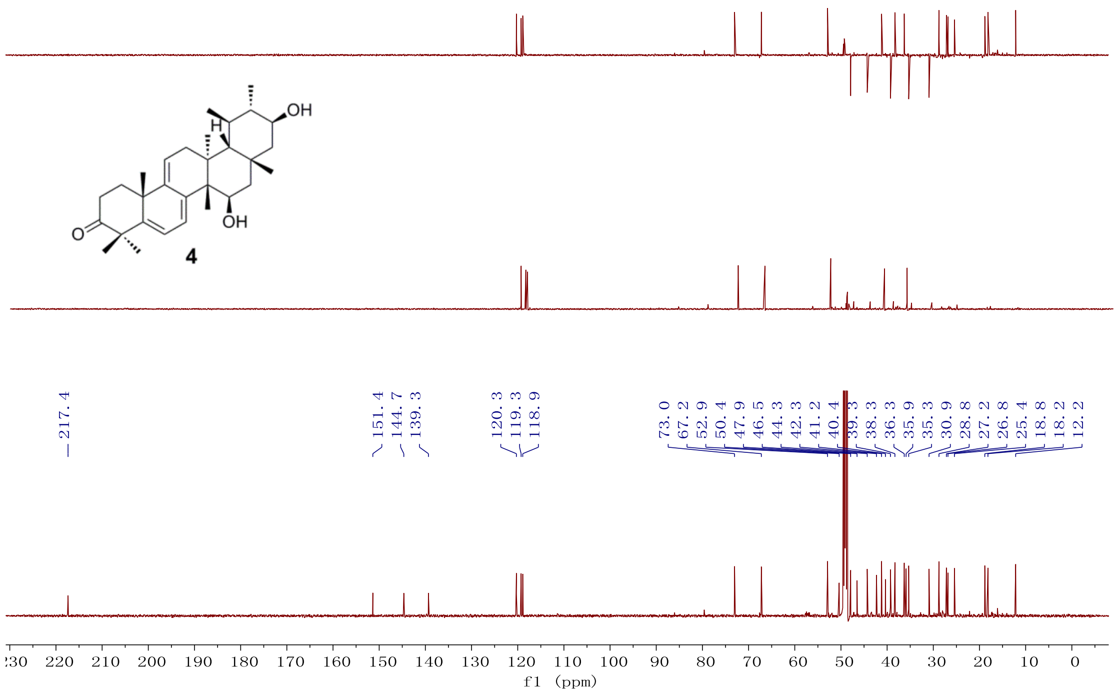

Figure S36. The ^{13}C NMR spectrum and DEPT spectrum of compound **4** in CD_3OD

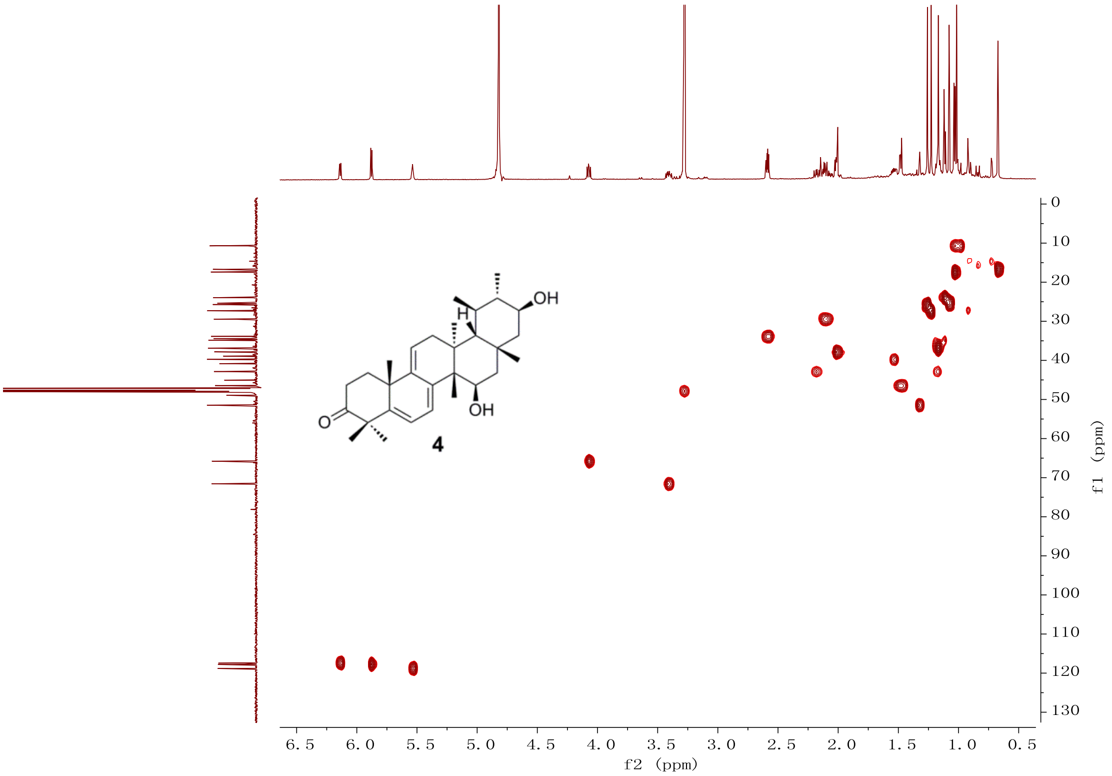

Figure S37. The HSQC spectrum of compound **4** in CD_3OD

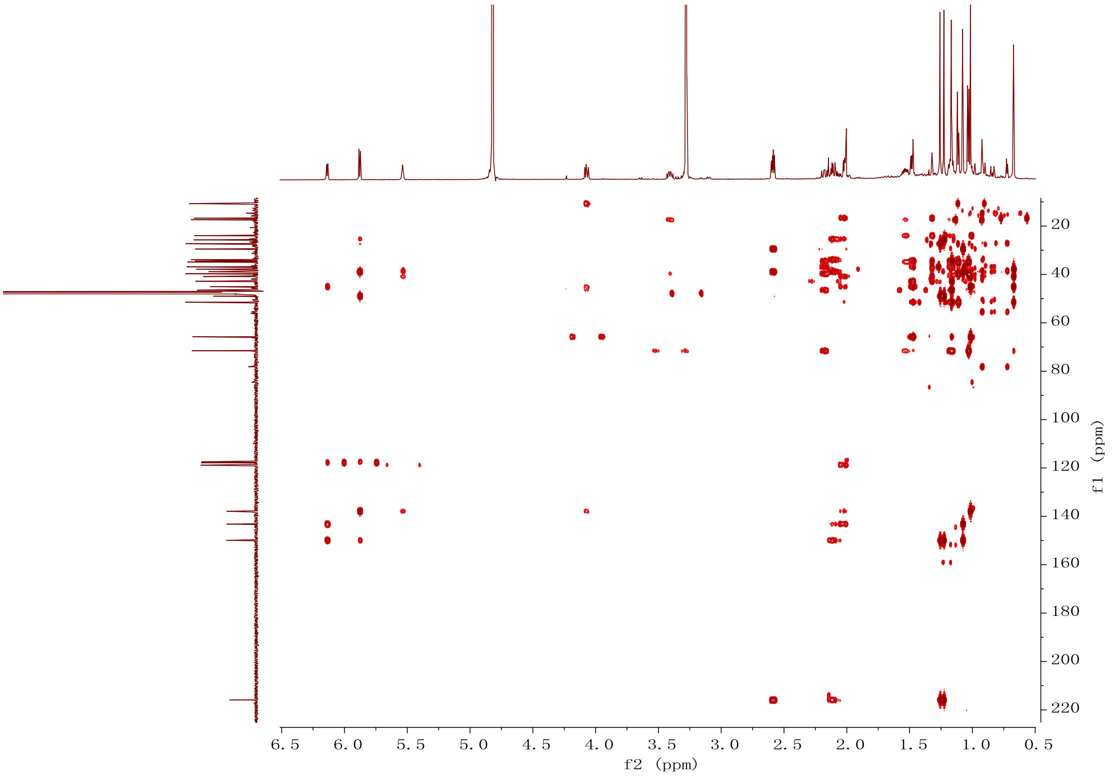

Figure S38. The HMBC spectrum of compound **4** in CD_3OD

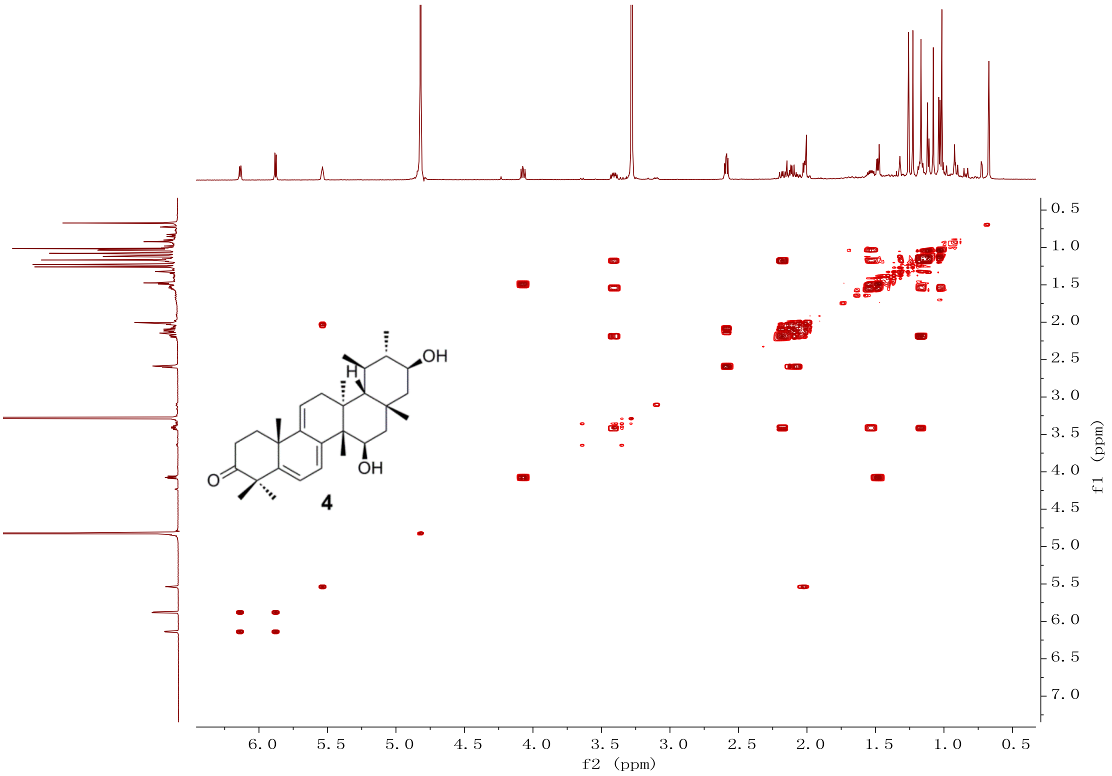

Figure S39. The ^{1}H-^{1}H COSY spectrum of compound **4** in CD_3OD

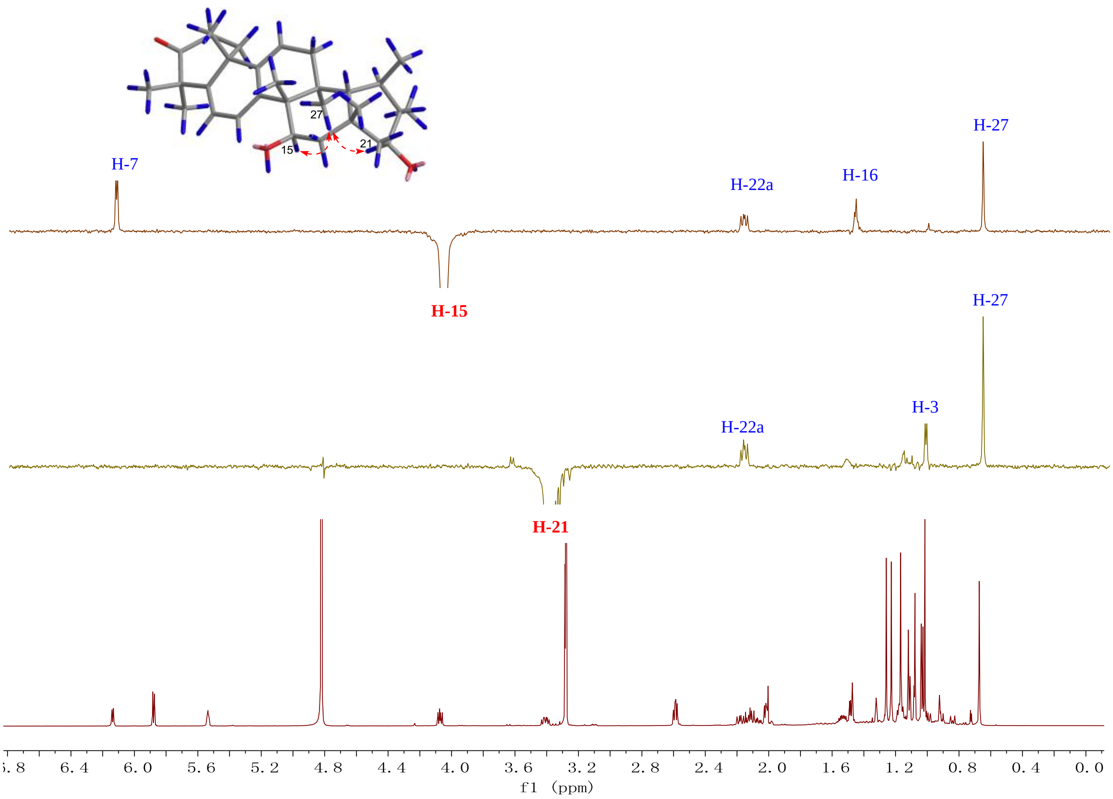

Figure S40. The NOE spectrum of compound **4** in CD_3OD
